# Kazakh Tobet dogs in the genomic landscape: refining the history of livestock guardian breeds

**DOI:** 10.1186/s12915-025-02344-2

**Published:** 2025-08-05

**Authors:** Anastassiya Perfilyeva, Kira Bespalova, Yelena Kuzovleva, Mamura Begmanova, Almira Amirgaliyeva, Olga Vishnyakova, Inna Nazarenko, Sergey Bespalov, Assel Zhaxylykova, Arailym Yerzhan, Kanagat Yergali, Yuliya Perfilyeva, Rustam Mussabayev, Zhassulan Zhaniyazov

**Affiliations:** 1 Laboratory of Molecular Genetics, Institute of Genetics and Physiology, Almaty, 050060 Kazakhstan; 2 Department of Cynology, Republican Federation of Public Associations of Hunters and Hunting Societies “Kansonar”, Almaty, 050008 Kazakhstan; 3 Department of Cynology, Republican Federation of Public Associations of Hunters and Hunting Societies “Kansonar”, Astana, 020000 Kazakhstan; 4 Laboratory of Theriology, Institute of Zoology, Almaty, 050060 Kazakhstan; 5 Laboratory of Molecular Immunology and Immunobiotechnology, M.A. Aitkhozhin’s, Institute of Molecular Biology and Biochemistry, Almaty, 050012 Kazakhstan; 6 Laboratory of Informational Processes Analysis and Modelling, Institute of Information and Computational Technologies, Almaty, 050000 Kazakhstan; 7 Laboratory of Population Genetics, Institute of Genetics and Physiology, Almaty, 050060 Kazakhstan; 8 Department of Chemical and Biochemical Engineering, Satbayev University, Almaty, 050013 Kazakhstan; 9 Faculty of Biology and Biotechnology, Al-Farabi Kazakh National University, Almaty, 050040 Kazakhstan

**Keywords:** Kazakh Tobet, Livestock guarding dog, Genomic characterization, Genetic diversity, Phylogeny, Signatures of selection

## Abstract

**Background:**

Despite the growing body of research on livestock guarding dog (LGD) breeds, Central Asian LGDs remain significantly underrepresented in genetic studies. Additionally, while some research has explored the genetic basis of selective traits in LGDs, significant gaps remain. By investigating the genetic diversity, evolutionary history, and adaptive traits of the Kazakh Tobet, a Central Asian LGD breed from Kazakhstan, we aim to contribute to a deeper understanding of the genetic basis that characterizes LGD breeds.

**Results:**

We analyzed the genetic relatedness of Kazakh Tobet dogs by combining SNP data from 90 unrelated Kazakh Tobets with publicly available SNP data from 124 dogs from 17 LGD breeds, 1753 dogs from 140 non-LGD breeds, 551 free-ranging dogs, and 16 wolves. The Kazakh Tobets exhibit considerable genetic diversity. Phylogenetic and PCA analyses suggest a common genetic origin with LGDs from Central and West Asia, which was complicated by gene flow from European LGDs. Signs of introgression from East Asian and Arctic non-LGDs may indicate a historical or modern gene exchange affecting the Kazakh Tobet lineage. Genetic clustering with free-ranging dogs supports the idea that open breeding systems do not compromise the functional integrity of LGD traits. Selection scans identified eight genomic regions with 127 genes associated with cognitive function, immune response, and metabolic regulation, suggesting the evolutionary importance of these traits in Kazakh Tobets.

**Conclusions:**

This study provides new insights into the genetic history of LGD breeds and their connections to the broader genetic landscape of non-LGD breeds.

**Supplementary Information:**

The online version contains supplementary material available at 10.1186/s12915-025-02344-2.

## Background

Livestock guarding dogs (LGDs) have long served as indispensable protectors of livestock in a variety of pastoral contexts, from nomadic migrations to settled agricultural communities across Eurasia. While it was previously believed that modern LGDs descended from a single ancestor, a more recent study has revealed a more complex evolutionary history [1]. It has been suggested that LGDs have multiple origins and that there was extensive gene flow between the breeds caused by the historical migrations of livestock and humans. Furthermore, crossbreeding with free-ranging dogs disproves the assumption that reproductive isolation is necessary to maintain the specialized working traits of LGDs. Despite considerable progress in understanding the origin and genetic diversity of LGDs, research has largely focused on populations from Europe and from East, West, and South Asia [1–5], with Central Asian breeds being significantly underrepresented. However, genetic studies on domestic animals have consistently shown that Central Asia was a hub for the evolution of domesticated species and an important intersection of genetic migration routes [6–8]. Given the historical importance of the region and the distinct adaptive traits of its breeds, it is imperative that research be directed towards the genetic characterization of Central Asian LGD breeds. Such studies are crucial to gain a more holistic understanding of the evolutionary processes that have shaped the LGD breeds, particularly regarding their complex interactions with the environmental, ecological, and human-induced factors in the region.

The Kazakh Tobet is a large, ancient Central Asian LGD breed native to Kazakhstan. Historically, Kazakh Tobets were used for guarding livestock from predators and robbers, protecting nomadic settlements and guarding and escorting caravans. These tasks required exceptional adaptability to the natural environment. The harsh continental climate of Kazakhstan, characterized by severe winters, scorching summers, and extreme temperature fluctuations, had a decisive influence on the resilience and endurance of the Kazakh Tobet. These dogs were able to survive “dzut” (mass deaths of livestock due to extreme winter conditions) and long periods of drought, living alongside nomadic herders in remote grazing areas. Unlike many other working dogs, Kazakh Tobets were often kept in the wild, independently guarding livestock across vast landscapes with minimal human supervision. They were also capable of traveling long distances alongside migrating herds, a trait essential to their role in traditional nomadic pastoralism. The intense environmental pressures of the Kazakh Tobet habitat led to the development of exceptional endurance, resilience to food and water scarcity, and heightened senses that ensured their survival and effectiveness as livestock guardians.

Today, a Kazakh Tobet that has retained its working traits has become increasingly rare, and the breed is under serious threat of extinction. Over the last century, socio-economic changes such as the transition from a nomadic to a sedentary lifestyle, the decline of sheep farming, wars, and political instability have hit the breed hard. In addition, other threats — such as dog fighting, infectious diseases, and poisoning from rodent control programmes — have also contributed to their decline. However, the greatest challenge remains uncontrolled breeding and crossbreeding with non-LGD breeds, which has led to an erosion of breed integrity and the loss of specialized working traits [9]. These factors have undoubtedly affected the population structure and diversity of the Kazakh Tobet, necessitating a comprehensive assessment of the current state of the breed. Our previous genetic analyses, including microsatellite markers and whole-exome sequencing (WES) on a small sample, provided initial insights into the genetic composition and relatedness of Kazakh Tobets [10]. This study revealed a high genetic diversity within the breed and indicated admixture with other LGD breeds, as phylogenetic analyses could not place the Kazakh Tobets into a clearly defined group. However, further genomic analysis is needed to deepen our understanding of this breed.

We conducted a comprehensive comparison to obtain a detailed picture of the genetic background of Kazakh Tobet dogs, including both LGD and non-LGD modern breeds, as well as free-ranging dogs and wolves. The most diverse dataset possible was compiled to allow a comprehensive assessment of common ancestry, possible recent admixture, basal lineage contributions, and genetic distinctiveness of the Kazakh Tobet. In addition, the extensive dataset generated in this study for the Kazakh Tobet enabled an investigation of the genomic regions potentially associated with the adaptive traits of the LGD. By examining the genetic structure of the Kazakh Tobet, we aimed to contribute to a better understanding of the evolutionary history of Central Asian LGDs and provide baseline data that can support future conservation and management efforts of the breed.

## Results

### Genetic diversity and population structure of Kazakh Tobet dogs and their genetic relationships with other LGD breeds

We evaluated the genetic diversity of Kazakh Tobet dogs from four regions of Kazakhstan and from Mongolia by calculating the observed heterozygosity (Ho), expected heterozygosity (He), polymorphism information content (PIC), and inbreeding coefficient (Fis) using the Kazakh Tobet dataset (Table 1). Ho values did not vary too much between regions and ranged from 0.348 in Kazakh Tobet dogs from West Kazakhstan to 0.369 in South Kazakhstan. The He values slightly exceeded the Ho value only in Kazakh Tobet dogs from South and North Kazakhstan, where the He values were 0.371 and 0.360, resulting in positive Fis values (0.005 and 0.026, respectively). Kazakh Tobets from other regions had negative Fis values. PIC values ranged from 0.159 in dogs from Mongolia to 0.219 in dogs from the South region.

**Table 1:** Genetic variability in Kazakh Tobet dogs evaluated with SNP markers

Region	Ho	He	PIC	Fis
South Kazakhstan	0.369 ± 0.131	0.371 ± 0.120	0.219 ± 0.045	0.005 ± 0.137
North Kazakhstan	0.350 ± 0.155	0.360 ± 0.131	0.213 ± 0.053	0.026 ± 0.234
East Kazakhstan	0.368 ± 0.184	0.356 ± 0.138	0.210 ± 0.059	− 0.034 ± 0.287
West Kazakhstan	0.348 ± 0.260	0.315 ± 0.173	0.186 ± 0.089	− 0.109 ± 0.408
Mongolia	0.365 ± 0.353	0.277 ± 0.204	0.159 ± 0.114	− 0.322 ± 0.483
Total	0.364 ± 0.123	0.373 ± 0.119	0.220 ± 0.043	0.023 ± 0.109

Compared to the genetic diversity levels of other LGD breeds in the LGD dataset (Additional file 1: Table S1), the Ho of the entire Kazakh Tobet population (0.364) fell within the range of breeds such as the Pshdar dog (0.363), Sarplaninac (0.363) and Kangal (0.353). It exceeded the values observed in breeds with the lowest heterozygosity, such as the Rafeiro of Alentejo (Ho = 0.281) and the Great Pyrenees (Ho = 0.243) and approached the highest Ho value found in the Turkmen Alabay (Ho = 0.375). The He and PIC values in Kazakh Tobet were the highest observed in other LGD breeds (0.373 and 0.22, respectively). Kazakh Tobet also showed a generally low inbreeding value, albeit with a slightly positive Fis (0.023), while most of the breeds showed negative inbreeding values. Nevertheless, the inbreeding values for Kazakh Tobet dogs from East Kazakhstan, West Kazakhstan, and Mongolia also showed negative values, as mentioned above.

**Fig. 1:**
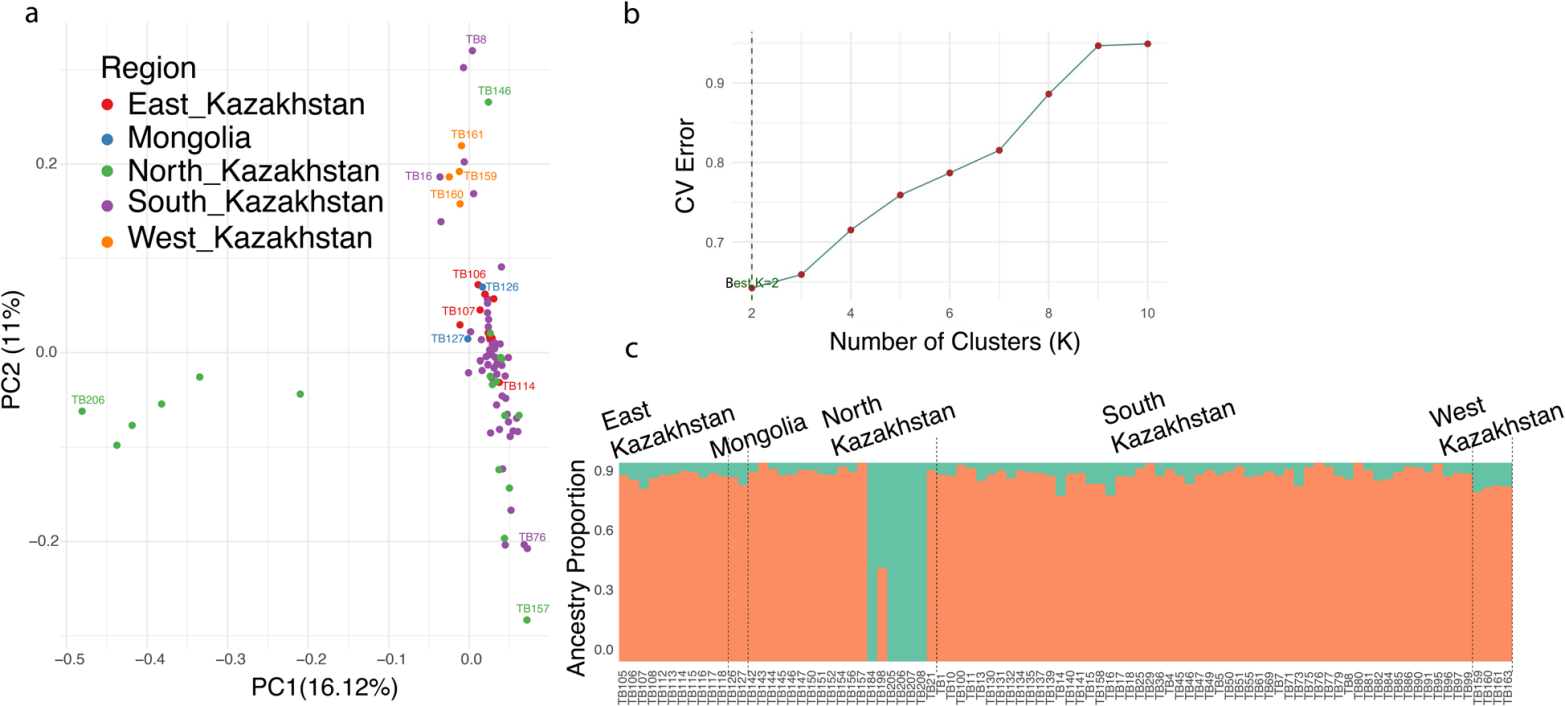
Genetic structure of 90 unrelated Kazakh Tobet dogs from four regions of Kazakhstan and Mongolia. **a** PCA plot. **b** CV errors for different *K* values. **c** Admixture plot for best *K* = 2

To analyze the population structure of Kazakh Tobet dogs, principal component analysis (PCA) was performed (Fig. 1a). The Kazakh Tobet dogs from East Kazakhstan, Mongolia, and most of the dogs from South Kazakhstan showed genetic similarities, as evidenced by their close clustering in the PCA plot. However, a subset of dogs from South Kazakhstan showed significant genetic variation along PC2. The dogs from North Kazakhstan showed a more diverse genetic structure in both PC1 and PC2, with certain individuals clustering with samples from other regions, while others formed a distinct subgroup. In addition, the dogs from West Kazakhstan formed a separate group clustered with some samples from South Kazakhstan.

While PCA revealed some genetic differentiation, the genetic structure of Kazakh Tobet dogs from different regions, except for those from North Kazakhstan, appeared to be largely similar in the admixture analysis. The lowest cross-validation (CV) error was observed for cluster *K* = 2 (0.6422, Fig. 1b). At this cluster level, only some Kazakh Tobet dogs from North Kazakhstan showed different genetic profiles compared to all other dogs (Fig. 1c).

To minimize potential bias due to uneven regional sampling, we also conducted PCA and admixture analyses with a dataset restricted to a maximum of five dogs per region. Both analyses resulted in clustering patterns that were consistent with the full dataset (Additional file 2: Fig. S1).

**Fig. 2:**
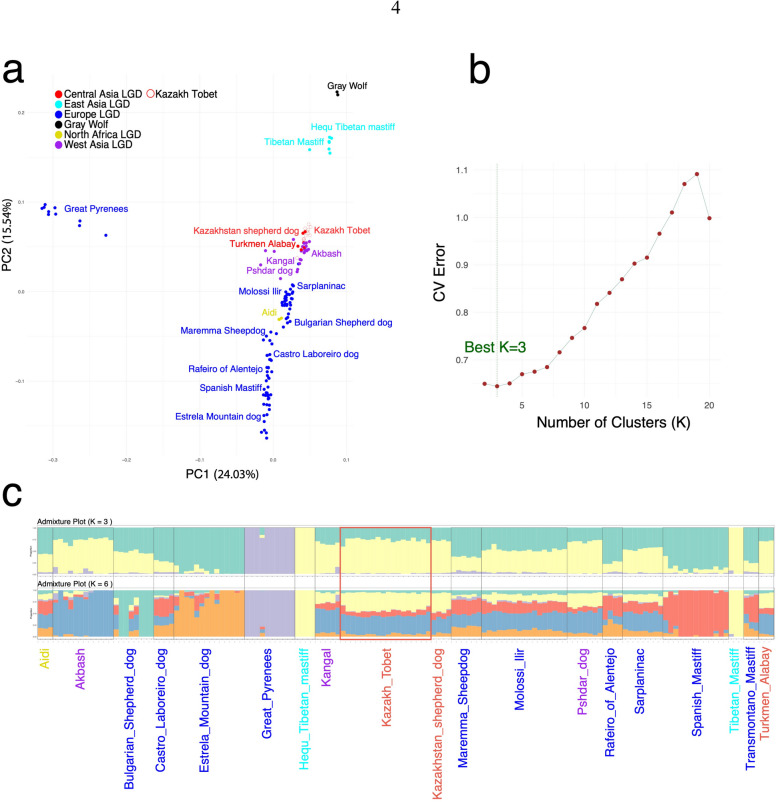
Comparative genetic structure of Kazakh Tobet dogs and other LGD breeds. **a** PCA plot. **b** CV error for different K values. **c** Admixture plots at cluster levels *K* = 3 and *K* = 6

We investigated the genetic relationship of Kazakh Tobet dogs with other LGD breeds by analyzing an LGD dataset that includes breeds from Africa, different regions of Asia, and Europe. PCA analysis revealed that Kazakh Tobet dogs were most closely clustered with LGD breeds from Central and West Asia, including Turkmen Alabay, Kangal, Pshdar dogs, and Akbash (Fig. 2a). Interestingly, all LGD breeds, except for the Great Pyrenees, formed a relatively tight cluster on the PCA plot. The admixture analysis confirmed these clustering patterns. The optimal K value for this dataset was 3 (CV error = 0.6443, Fig. 2b). At this level of clustering, only the Great Pyrenees formed distinct clusters, while the other LGD breeds had predominantly similar profiles (Fig. 2c). When the number of clusters was increased (*K* = 6), the Kazakh Tobets showed a similar distribution of color clusters as breeds from West and Central Asia, e.g., the Kangal, the Pshdar dog, and the Turkmen Alabay, confirming the PCA results. Some European breeds also showed similar admixture patterns (Molossi Ilir, Sarplaninac). Kazakhstan Shepherd dogs (Kazakh Tobet dogs from Xinjiang) showed identical genetic profiles to the Kazakh Tobet dogs from Kazakhstan. Remarkably, the Kazakh Tobet sample used in this dataset included dogs from different regions with the highest breed conformation scores, and their genetic profiles remained remarkably consistent.

**Fig. 3:**
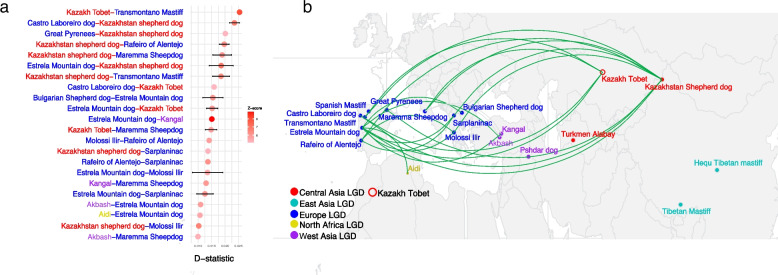
Signatures of gene introgression between LGD breeds based on D-statistics analysis. **a** Pairwise comparisons of LGD breeds are shown on the left. Corresponding Z-scores are shown as points on the right, with horizontal bars representing standard deviations (SD). The points are shaded in varying levels of transparency to reflect the intensity of the *Z*-score, with higher opacity (e.g., darker red) indicating stronger statistical support for gene flow. **b** Significant introgression events between LGD breeds visualized on a geographical map

The D-statistics were further used to examine patterns of historical gene flow between LGD breeds (Fig. 3a and b, Additional file 1: Table S2). A number of significant introgression signals were identified. Kazakh Tobet dogs showed signs of gene flow with several European breeds, such as the Estrela Mountain Dog, Maremma Sheepdog, Transmontano Mastiff, and Castro-Laboreiro dog. A similar pattern was observed for the Kazakhstan Shepherd Dog, which shared genetic signals with the Maremma Sheepdog, Rafeiro of Alentejo, Sarplaninac, etc.

**Fig. 4:**
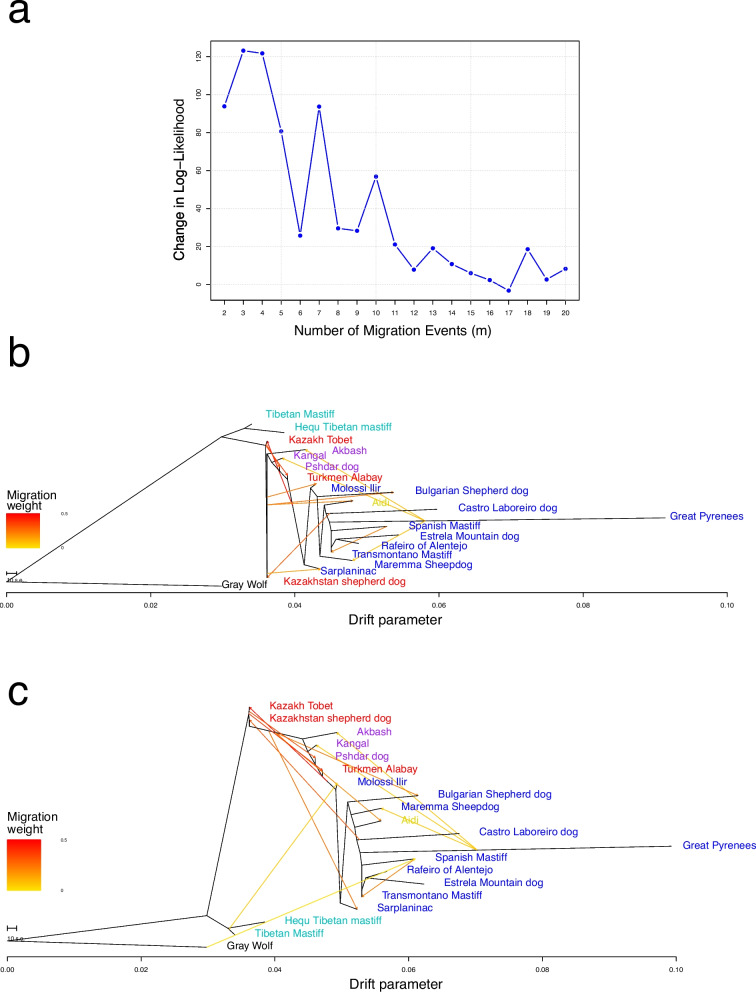
Signatures of genomic introgression among LGD breeds based on the results of the TreeMix analysis.** a** Log-likelihood curve, which tests up to 20 possible migration events. **b**, **c** Maximum likelihood phylogeny inferred with TreeMix analysis, with the gray wolf as root. The model includes 12 (**b**) and 13 (**c**) migration events. TreeMix-inferred migration events are represented by arrows showing the direction of gene flow from the donor to the recipient population, with colors reflecting the weight of migration. Branch lengths are proportional to the estimated genetic drift

We further investigated both the phylogenetic relationships and the migration events by performing a TreeMix analysis. Of the 20 migration events tested, migration events 12 and 13 were selected as optimal migration events, as the inclusion of further migration events led to an ever smaller increase in the likelihood (Fig. 4a). In addition to the migration patterns observed between the different LGD breeds, the analysis revealed significant introgression events between Kazakh Tobet dogs and the Turkmen Alabay and Pshdar dogs (Fig. 4b). In the model with 13 migration events, gene flow was also observed between the Kazakh Tobet and the Castro Laboreiro dog, which had previously been revealed by the D-statistics analysis (Fig. 4c).

### Global genetic relationships of Kazakh Tobet dogs with other dog breeds and free-ranging dogs

**Fig. 5:**
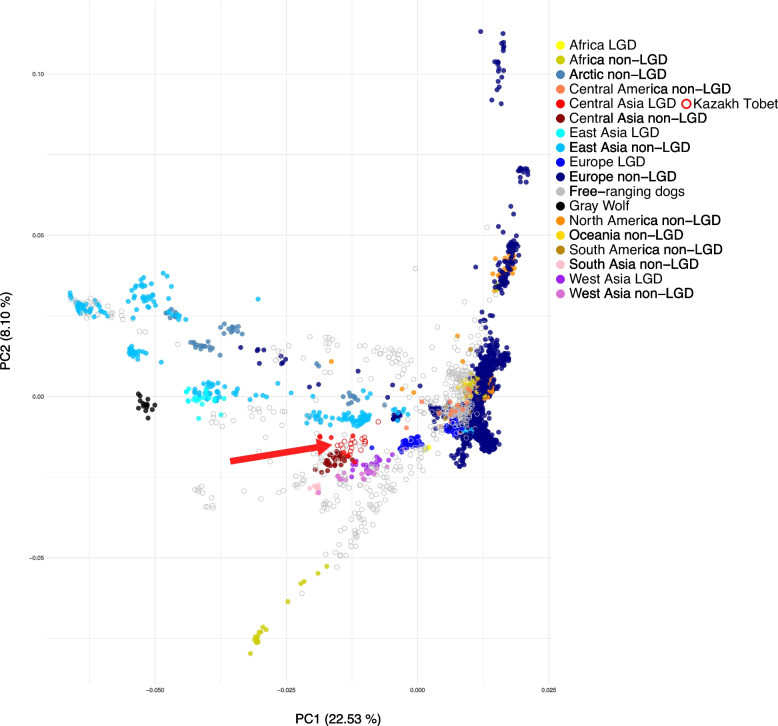
PCA of Kazakh Tobet dogs in a global context with other 17 LGD breeds, 140 non-LGD breeds, free-ranging dogs, and wolves

For a comprehensive understanding of the genetic structure of Kazakh Tobet dogs in a global context, a PCA was performed with a combined dataset of LGD, non-LGD, and free-ranging dogs (Fig. 5). The PCA revealed ten major axes of genetic variation, with the first three components accounting for 22.53%, 8.10%, and 7.06% of the total variance, respectively, based on the full set of eigenvalues. Among LGD breeds, Kazakh Tobet dogs were consistently closely clustered with LGD breeds from Central and West Asia. Within the non-LGD breeds, Kazakh Tobet dogs, along with Kazakhstan Shepherd dogs, showed genetic proximity to the sighthounds from Central Asia, the Kazakh Tazy and Taigan (Additional file 2: Fig. S2). Among the non-LGD breeds from other regions, East Asian and Arctic breeds showed the closest genetic affinity to the Kazakh Tobet. Finally, the Kazakh Tobet dogs were found to be genetically closely related to free-ranging dogs.

**Fig. 6:**
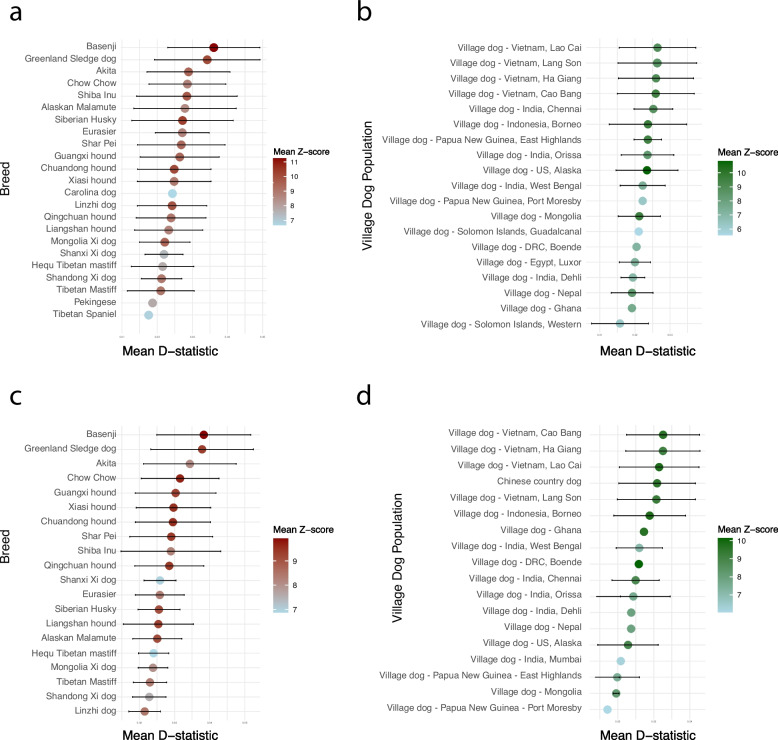
Gene flow of Kazakh Tobet dogs and Kazakhstan Shepherd dogs in the global context of dog populations based on D-statistics. The left panel in each figure shows the breed or population involved in significant gene flow events. Corresponding Z-scores are shown as points on the right, with horizontal bars representing SD. Points are color-coded by *Z*-score intensity, with color gradients indicating the strength of statistical support for gene flow. **a** Gene flow of Kazakh Tobet dogs with other LGD and non-LGD breeds. **b** Gene flow of Kazakh Tobet dogs with free-ranging dogs. **c** Gene flow of Kazakhstan Shepherd dogs with other LGD and non-LGD breeds. **d** Gene flow of Kazakhstan Shepherd dogs with free-ranging dogs

The D-statistic was then used to evaluate the genetic relationships between Kazakh Tobet dogs and non-LGD breeds. The results revealed a consistent pattern of gene flow between the Kazakh Tobet and various Arctic and East Asian non-LGD breeds (Fig. 6a and c). In the combined dataset, the D-statistic also showed a notable signal of gene flow between Kazakh Tobet dogs and East Asian LGD breeds, such as the Tibetan Mastiff and Hequ Tibetan Mastiff. The lack of this signal in the more restricted LGD dataset is likely due to the overall genetic similarity between LGD breeds, which may reduce the sensitivity of the D statistic in detecting introgression events. In addition, both Kazakh Tobet and Kazakhstan Shepherd dogs showed signals of introgression with free-ranging dog populations from geographically distant regions — including India, Vietnam, Indonesia, New Guinea, the Pacific Islands, and others (Fig. 6b and d).

An admixture analysis was performed to confirm the patterns identified in the PCA and D-statistics results (Additional file 2: Fig. S3). This analysis was extended to non-LGD molossoid breeds from Europe and America — such as American Pit Bull Terrier, American Staffordshire Terrier, Boxer, Bullmastiff, Cane Corso, Great Dane, Staffordshire Bull Terrier, Rottweiler, and Saint Bernard — with which Kazakh Tobet dogs may have been crossbred without strict breeding control to improve traits such as strength, body size, and fighting ability. The Kazakh Tobet did not form a distinct cluster in the admixture plots when exploring its genetic relationships with Central Asian sighthound breeds as well as Arctic and East Asian non-LGD breeds (Additional file 2: Fig. S3a–d). The analysis revealed a clear shared genetic component between the Kazakh Tobet and the Central Asian sighthounds, the Kazakh Tazy and Taigan. Additionally, evidence of partial genetic admixture with Arctic and East Asian non-LGD breeds was observed in the Kazakh Tobet. Remarkably, the Tobet individuals also showed partial admixture with non-LGD molossoid breeds (Additional file 2: Fig. S3e–f). Interestingly, the Kazakh Tobet dogs from the North region of Kazakhstan lacked such admixture signals and showed a very homogeneous genetic pattern.

**Fig. 7:**
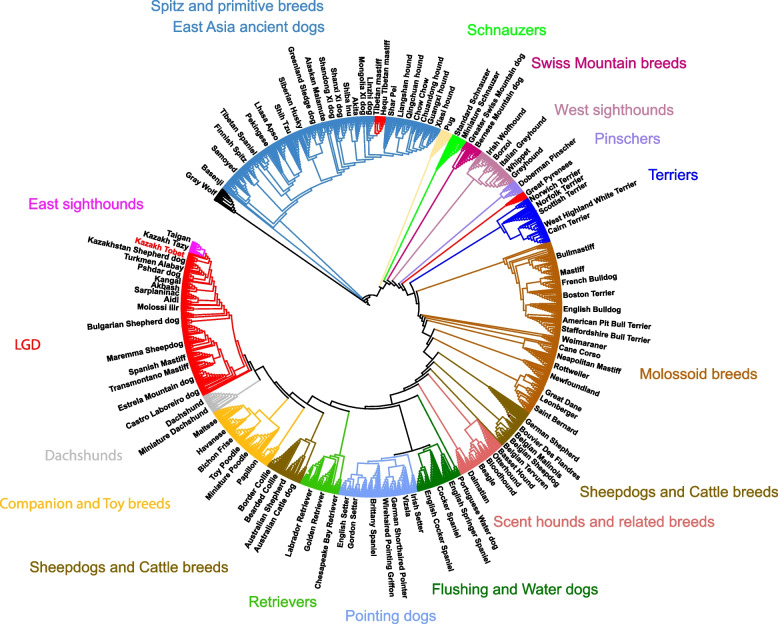
NJ tree, rooted with wolves. Different colors were used to differentiate breed groups, with LGD breeds highlighted in red

A neighbor-joining (NJ) tree was constructed to evaluate the phylogenetic relationships of the Kazakh Tobet breed with both LGD and non-LGD breeds (Fig. 7). The Kazakh Tobet and Kazakhstan Shepherd dog showed a close genetic relationship and were grouped within the broader LGD breed group. They showed the strongest genetic relatedness to Central (Turkmen Alabay) and West Asian breeds (Pshdar dog and Kangal), confirming the results of previous analyses. Among the non-LGD breeds, the Kazakh Tobets were phylogenetically most closely related to the Central Asian sighthounds, the Kazakh Tazy and Taigan.

### Selection signatures of Kazakh Tobet dogs

**Fig. 8:**
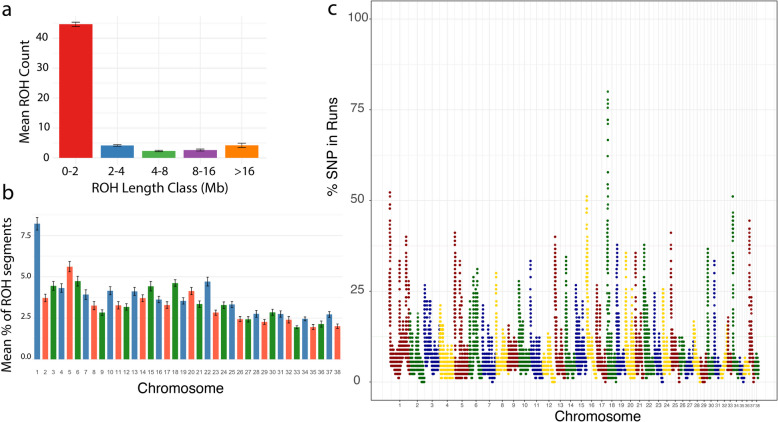
Characteristics of the ROH of Kazakh Tobet dogs. **a** The average number of ROH per length class. **b** The average proportion of ROH per CFA. **c** Manhattan plot of the distribution of ROH

Runs of homozygosity (ROH), integrated haplotype score (iHS) and number of segregating sites by length (nSL) tests were used to identify selection signals for 90 unrelated Kazakh Tobet dogs. A total of 4820 ROH were identified. The majority of ROH were short, with 4017 segments (83.34%) in the 0–2 Mb range, followed by 375 segments (7.78%) in the 2–4 Mb range. Medium-length ROH were less common, with 160 segments (3.32%) in the 4–8 Mb range and 113 segments (2.34%) in the 8–16 Mb range. The longest ROH (> 16 Mb) were the least frequent, accounting for 155 segments (3.22%). The average number of ROH segments per length class is shown in Fig. 8a. The highest proportion of ROH was observed on *Canis* *famiaris *autosome (CFA) 1, followed by CFA 5, 22, and 18. In contrast, the lowest proportion was found on CFA 35 and 38 (Fig. 8b).

**Fig. 9:**
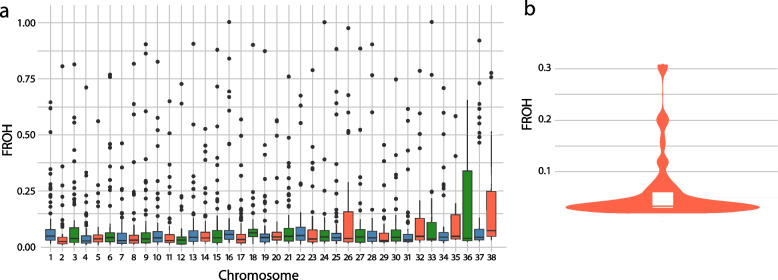
Characteristics of the FROH of Kazakh Tobet dogs. **a** FROH by CFA. **b** Distribution of FROH

The highest average level of ROH-based inbreeding (FROH) were found on CFA 36 (Fig. 9a). Variability in genomic inbreeding was observed in the dogs (Fig. 9b and Additional file 1: Table S3). While most dogs had moderate FROH values between 0.02 and 0.07, a small subgroup showed significantly elevated values of more than 30%. Individuals from North Kazakhstan (TB184, TB205–TB208), which were clustered separately in both the PCA and admixture analyses, consistently had some of the highest FROH values in the dataset.

Four extreme runs of homozygosity islands (eROHi) were identified on chromosomes CFA 1, 16, 18, and 34 (Fig. 8c). The most prominent pattern was observed on CFA 18, where an overlapping ROH region was present in 78 Kazakh Tobet dogs (Table 2). In total, 78 SNPs (Additional file 1: Table S4) and 17 genes were located within these four eROHi regions, including four candidate genes with known functional significance: *GALR1*,* MBP*,* CNTNAP2*, and *TRIO.*

**Table 2:** Characteristics of the eROHi of the Kazakh Tobet dogs

*N*	CFA	nSNP	From	To	Size (kb)	nDog	Genes
1	1	4	2,550,379	3,041,620	491,241	47	*GALR1*, *MBP*
2	16	2	2,262,752	2,292,238	29,486	46	*CNTNAP2*
3	18	22	3,319,077	4,837,968	1,518,891	78	
4	34	3	624,977	708,823	83,846	46	*TRIO*

**Fig. 10:**
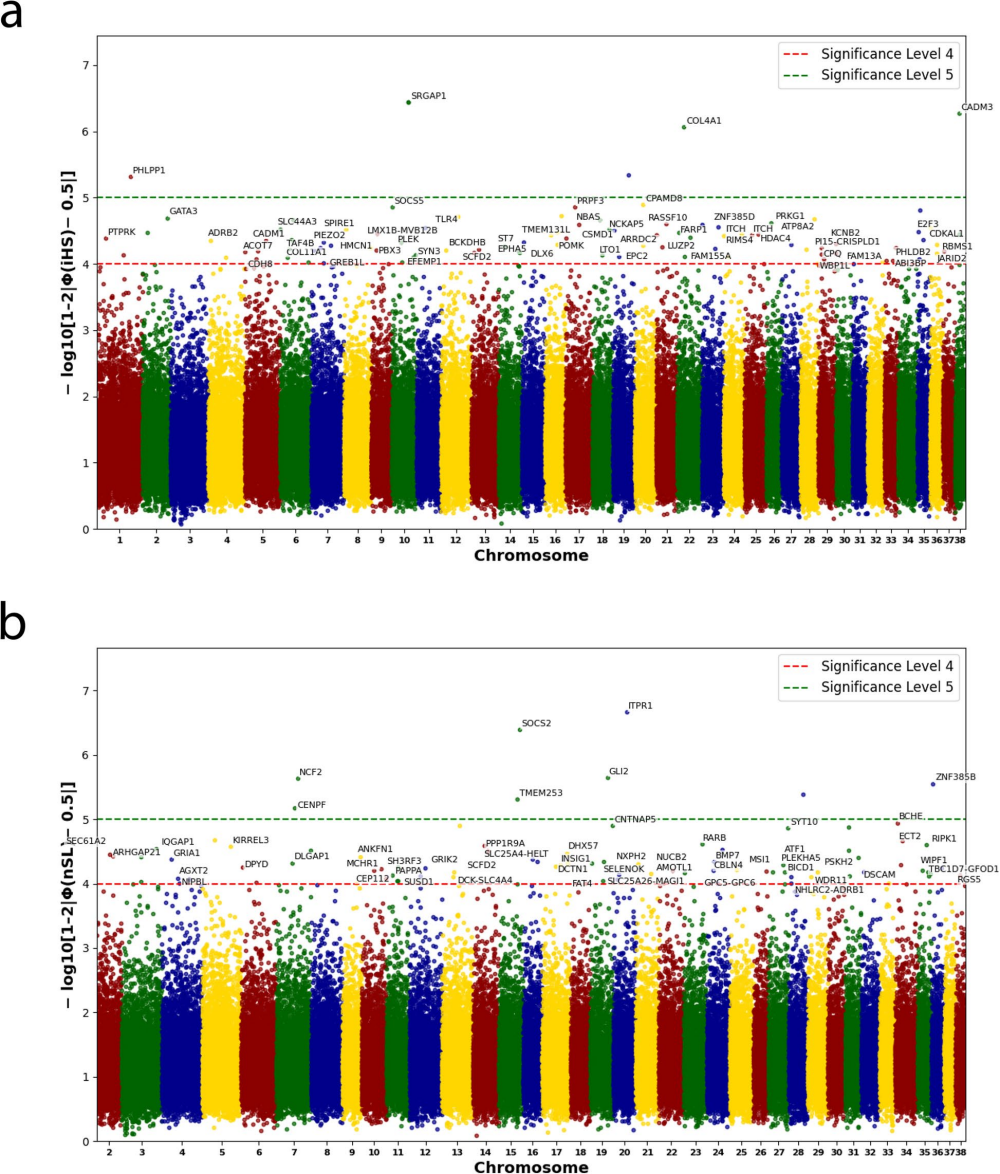
Manhattan plots of the selection scan results. **a** iHS test. **b** nSL test

A total of 187 autosomal outlier SNPs with significant evidence of selection (− log10(*p*-value) > 4) were identified by summing the results of the iHS and nSL tests (Fig. 10 and Additional file 1: Table S4). Among these SNPs, 250 genes were mapped, of which 123 have a known functional significance.

Four genomic regions in four CFAs, each containing at least two SNPs located in relative proximity, were identified as the most likely candidates for positive selection (Table 3).

**Table 3:** Signatures of selection identified in the iHS and nSL test

*N*	CFA	nSNP	From	To	Size (kb)	Genes
1	10	2	6,757,374	6,761,621	4,247	*SRGAP1*
2	13	2	45,682,092	45,713,065	30,973	*SCFD2*
3	24	2	23,491,402	23,509,687	18,285	*ITCH*
4	38	2	22,770,801	22,804,623	33,822	*CADM3*

The annotations of 127 genes with known functional significance for the identified selection signals using ROH, iHS, and nSL tests are listed in Additional file 1: Table S5. The functional annotation of the candidate genes revealed two Gene Ontology (GO) terms and 11 Kyoto Encyclopedia of Genes and Genomes (KEGG) pathways with nominally significant enrichment (*p* < 0.05; Additional file 1: Table S6), although none of them reached significance after Bonferroni correction. Both GO terms and three of the KEGG pathways were associated with neuronal signaling and cognitive function. These included glutamatergic synapse (GO:0098978), postsynaptic specialization (GO:0099572), glutamatergic synapse (cfa04724), long-term depression (cfa04730) and circadian entrainment (cfa04713). The genes *DLGAP1*, *GRIA1*, *GRIK2*, and *PLEKHA5* were significantly associated with this term and the KEGG pathways. Four other KEGG pathways were associated with intracellular signaling, secretion, and intercellular communication, including salivary secretion (cfa04970), gap junction (cfa04540), and cGMP–PKG signaling (cfa04022), enriched for *ADRB1, ITPR1*,* PRKG1*, and *SLC25A4*. The remaining signaling pathways were associated with immune and metabolic processes, such as the formation of extracellular traps for neutrophils (cfa04613; *HDAC4, NCF2, SLC25A4, TLR4*), regulation of lipolysis in adipocytes (cfa04923; *ADRB1*,* PRKG1*) and pyrimidine metabolism (cfa00240; *DCK*,* DPYD*). Two disease-related pathways — inflammatory bowel disease (cfa05321; *GATA3, TLR4*) and cancer pathways (cfa05222; *COL4A1*,* E2F3*,* RARB*) — were also enriched.

## Discussion

### Genetic diversity and structure of the Kazakh Tobet dogs and their genetic relationships with other LGD breeds

This study represents the most comprehensive genetic analysis to date of both inter- and intra-breed relationships of the Central Asian LGD Kazakh Tobet. By using a SNP-based approach, we were able to assess genetic variation, phylogenetic relationships, admixture patterns, and identify potential selection signatures of the breed. The results indicate considerable variability of Kazakh Tobet dogs and a low degree of inbreeding, with some regional differences. These findings are consistent with our previous microsatellite-based study [10], although the microsatellite analysis yielded higher heterozygosity values (for Ho 0.78 *versus* 0.364 and for He 0.81 *versus* 0.373), likely due to the greater polymorphism of microsatellite markers, which generally capture a broader range of genetic variation. Despite this discrepancy, the diversity ratios obtained from SNP and microsatellite analyses were correlated, although the extent of variation may differ in some cases [11]. The genetic diversity observed in the Kazakh Tobet dogs was comparable to that of other LGD breeds analyzed with SNP arrays, with Ho values for LGD breeds ranging from 0.299 (Great Pyrenees) to 0.473 (Turkmen Alabay) and 0.479 (Aidi) [1, 2]. The Ho value of the Kazakh Tobet (0.363) is comparable to that of breeds such as the Akbash (0.365), Bulgarian Shepherd dog (0.37), Fonni’s dog (0.365) and Estrela Mountain dog (0.366). Our own LGD dataset confirms these results and shows that the genetic diversity of Kazakh Tobet dogs is comparable to that of other LGD breeds.

The genetic diversity and low inbreeding observed in Kazakh Tobet dogs are supported by ROH analysis, which provides valuable insight into the genetic history of the population. Longer ROH segments (> 10 Mb) indicate recent inbreeding, while shorter segments (1–4 Mb) reflect older inbreeding events [12]. The prevalence of shorter ROH segments in the Kazakh Tobets suggests distant inbreeding, possibly related to a larger historical effective population size and less isolation compared to populations with longer ROH segments [12]. In addition, analysis of FROH values further characterizes the inbreeding dynamics of the breed. Most dogs in the population had moderate FROH values, indicating a generally low level of inbreeding. However, a small group of dogs had significantly higher FROH values, above 30%, suggesting that specific inbreeding events occurred within the breed.

In the genetic structure of the Kazakh Tobet dogs, we observe a relatively homogeneous admixture pattern, with the exception of a subgroup from North Kazakhstan, which differs genetically from the other individuals both in the PCA and in the admixture analyses. Most of the dogs in this subgroup come from the same breeding kennel. The same homogeneity of genetic structure was also observed in the entire LGD dataset. At the optimal cluster level of *K* = 4, almost all breeds, including the Kazakh Tobets, showed a consistent admixture profile, except for the Great Pyrenees. Although previous microsatellite-based studies have reported higher intra- and inter-breed differentiation in LGDs [3, 10, 13], SNP-based analyses generally indicate a more homogeneous structure [5]. This trend is supported by a previous FST-based study that found low pairwise genetic differentiation between 36 LGD breeds, with the highest values observed in the Great Pyrenees [1].

As the number of clusters increased, the differentiation between breeds became more apparent, with the Kazakh Tobet dogs showing a pattern of admixture most similar to that of the LGD breeds from Central and West Asia. This result was also confirmed by PCA, which revealed a similar clustering of the Kazakh Tobets with breeds such as the Turkmen Alabay, Kangal, and Pshdar dog.

The observed low differentiation between all LGD breeds and especially the close genetic relationship between Kazakh Tobet dogs and LGD breeds from Central and West Asia can be attributed to several interacting factors. The hypothesis that LGD breeds have multiple origins suggests that these dogs evolved independently in different regions in response to the growing demands of livestock production and then spread widely [1]. The genetic relatedness observed in our study between the LGD breeds from Central and West Asia suggests that these breeds may share a common ancestral lineage, with regional adaptations shaping their current genetic structure. Alternatively, the observed clustering of these breeds could be due to a historical gene flow facilitated by the movement of herders and traders along pastoral routes and major trade networks, such as the Silk Road. The strategic location of Kazakhstan at the crossroads of Asia, combined with its historical role in trade and nomadic pastoralism, provides a compelling explanation for possible genetic interactions between LGD breeds in these regions. Our analysis further supports this hypothesis, as TreeMix analysis revealed a significant genetic flow between Kazakh Tobets and LGD breeds from Central and West Asia. In addition, D-statistics showed clear signals of introgression between both Kazakh Tobet and Kazakhstan Shepherd dogs (Kazakh Tobets from China) and several European LGD breeds. These results support previous studies demonstrating continuous gene flow between LGD populations facilitated by human and livestock migration [2, 3, 14]. A similar pattern of gene flow between Kazakhstan Shepherd dogs and distant European breeds was observed in previous D-statistics analyses [1]. In addition, historical records suggest that the Alans, a nomadic group that migrated westward from West Kazakhstan in the second century AD and reached the Iberian Peninsula in the fifth century AD, played a key role in the spread of a sighthound-mastiff cross called the Alaunt (or Alano), providing further evidence that dogs associated with the Steppe region were involved in the development of European breeds [15].

A third, still speculative explanation for the observed genetic similarity between Kazakh Tobet dogs and LGD breeds from Central and West Asia could be a parallel, independent selection pressure related to livestock guarding, possibly causing convergent genetic changes between these breeds. However, this hypothesis needs to be further investigated and validated. A fourth possible explanation for the observed genetic relatedness of Kazakh Tobet dogs with LGD breeds from Central and West Asia and for the signs of introgression with European breeds is contemporary gene flow through deliberate crossbreeding. The Kazakh Tobet breed has lacked a consistent and controlled breeding system in recent decades. According to kennel specialists, among all other LGD breeds, the Kazakh Tobet was most often crossbred with Central Asian Shepherd dogs and Caucasian Shepherd dogs. However, these breeds were not included in our analysis due to the unrepresentative samples in the publicly available genotyping data. Although crossbreeding with West Asian breeds such as the Akbash and Kangal may have occasionally occurred, these breeds are relatively rare in the country, making frequent crossbreeding less likely. The influence of such gene flow through intentional crossbreeding should be further explored.

### Global genetic relationships of Kazakh Tobet dogs with other dog breeds and free-ranging dogs

In addition, we evaluated the genetic relationships of the Kazakh Tobet in the context of a broader range of dog breeds, including LGD and non-LGD, as well as free-ranging dog populations. The PCA analysis further supported the common genetic background of the Kazakh Tobet dogs and the LGD breeds from Central and West Asia. Among the non-LGD breeds, the Kazakh Tobet dogs were clustered with the sighthound breeds of the ancient eastern group, such as the Kazakh Tazy and Taigan. The evolutionary link between LGDs and sighthounds has been well documented in previous studies [1, 16, 17] with an observation that LGDs and sighthounds from the same geographical regions exhibit more shared genetic background [1]. This characteristic clustering, which was also confirmed by our phylogenetic analysis, is particularly remarkable given the clear phenotypic and functional differences between LGDs and sighthounds — LGDs guarded livestock and sighthounds hunted game. A plausible explanation for this genetic similarity is gene flow and adaptation to shared ecological conditions. There is no doubt that gene flow between these breeds occurred in the past, which is confirmed not only by historical records, but also by our analysis, which found admixture between Kazakh Tobet dogs and these breeds. The Kazakhs called the mixed breed dog from Kazakh Tobet and Kazakh Tazy “duregei,” “drok.” In breeding practice, a Kazakh Tazy male was mated with a Kazakh Tobet female. The result was larger, Tazy-like dogs that retained some of the characteristics of the Kazakh Tobet. These hybrids were usually backcrossed with purebred Kazakh Tazy to enhance their hunting instincts and prey drive.

In addition, PCA analysis revealed a genetic relationship between the Kazakh Tobet dogs and ancient non-LGD breeds from an Arctic and East Asian region. This clustering was consistently supported by D-statistics and admixture analysis. Remarkably, significant gene flow signals were also detected between Kazakh Tobet dogs and geographically distant village dog populations from India, Vietnam, New Guinea, and the Pacific Islands. Several research studies can contribute to the understanding of these findings. Genomic evidence from Samoyedic-speaking communities such as the Nenets and Selkups suggests that these groups had genetic interactions with Steppe populations [18]. Archaeological finds, such as that from Ust-Polui in northwestern Siberia, demonstrate iron and bronze metallurgy as well as isolated artefacts such as glass beads that were likely brought from distant regions of the Steppe [19]. These proposed migrations and exchanges of material between people probably also involved dogs and contributed to the observed gene flow between Kazakh Tobet dogs and Arctic breeds. Based on the F4 ratio, it was assumed that about 40% of the ancestors of Steppe dogs originated from the Arctic lineage, represented by the Zhokhov Island dog [20]. Supporting this hypothesis, skeletal remains of dogs from the Eneolithic Botai culture in North Kazakhstan (ca. 3700–3100 BCE) were found to closely resemble the modern Samoyed in size and form, suggesting that ancestral Arctic-related dog lineages may have been present in this region as early as the fourth millennium BCE [21]. Furthermore, studies [22, 23] confirmed that modern and ancient dogs can be broadly categorized into three main groups: West Eurasian, East Asian, and Arctic/American. The East Asian lineage includes modern dogs from China, Vietnam, the Southeast Asian islands, dingoes, and New Guinea Singing dog. Many modern Chinese dogs have been shown to have clear evidence of admixture with a population related to the New Guinea Singing dog [22]. A whole-genome analysis of the New Guinea Singing dog and the dingo revealed that their ancestors originated in southern East Asia and migrated through the Southeast Asia islands before reaching Australia around 8300 years ago [24]. The Kazakh Tobet breed may have been formed at the intersection of Arctic/American and East Asian lineages, which explains its genetic links to ancient Arctic and East Asian breeds as well as to geographically distant free-ranging dogs. However, this hypothesis requires further validation given the debated sensitivity of the D-statistics and the possibility of false-positive results [25–27]. Nonetheless, the clear grouping of Kazakh Tobet dogs with well-established ancient breeds, as shown in the various analyses of this study, supports the conclusion previously based on haplotype analysis regarding the ancient origins of the breed [10].

Similar to the explanation of the genetic relationship between Kazakh Tobet dogs and other LGD breeds, we cannot exclude also the possibility that intentional crossbreeding could have an influence on the observed genetic patterns. According to kennel experts, crossbreeding with Arctic breeds may have been practiced in Kazakh Tobets to improve traits such as coat quality or endurance. It is plausible that more unconventional crossbreeding may have taken place, for example with East Asian breeds such as the Shiba Inu, Chow Chow, Shar-Pei, and other breeds. Interestingly, the D-statistics analysis showed introgression signals from the Eurasier, a breed of European origin, which itself is the result of a cross between the Chow Chow and a Spitz-type dog [16]. We also found evidence of admixture between Kazakh Tobet dogs and European and American mastiff-type non-LGD breeds. Interestingly, this admixture was not observed in individuals from a North Kazakhstan kennel, which instead showed consistently high rates of inbreeding based on ROH analysis. These patterns likely reflect genetically isolated breeding within the kennel. However, such high levels of inbreeding may have a negative impact on the health of the dogs. Further studies are needed to assess the effects of crossbreeding and inbreeding and their long-term consequences for the development of the breed.

Finally, the clustering of Kazakh Tobet dogs with free-ranging dogs confirms the widespread nature of this relationship within LGD breeds [17]. It also provides additional support for the previously proposed hypothesis that reproductive isolation is not required to maintain the high specialization of these dogs [1].

### Selection signatures of Kazakh Tobet dogs

As far as we know, this study represents the largest dataset ever compiled for a single LGD breed. It provides a valuable opportunity to uncover genomic regions that may be associated with adaptive traits in the Kazakh Tobet. Previous research has shown that the integration of multiple analytical approaches can significantly improve the accuracy in detecting selection signatures [28–30]. In this study, we applied multiple statistical methods, including haplotype-based tests (iHS and nSL) and ROH analysis, to identify genomic regions under selection. The haplotype-based method iHS effectively identifies intermediate selection signatures, where the selected allele has reached an intermediate frequency but is not yet fixed [31]. nSL, which is conceptually similar to iHS, is less sensitive to variations in the recombination rate and shows greater efficiency in detecting soft sweeps [32]. ROH analysis detects homozygous regions shared by a large proportion of individuals in a population and is characterized as eROHi genomic footprints indicative of selection events [33]. The main difference between haplotype-based tests and ROH analyses lies in the type of selection signals they detect. Haplotype-based tests are designed to detect recent positive selection events that leave distinct patterns of linkage disequilibrium decay around selected alleles. ROH analysis detects long homozygous regions that may result from historical selection pressure, inbreeding, or demographic events such as bottlenecks and genetic drift [34–37]. Possibly for these reasons, our results showed no overlap between the regions identified by these different methods. However, by integrating the iHS and nSL results, we were able to detect four genomic regions subject to selection, proving that these signals are biologically relevant and not statistical artifacts. We also identified four genomic regions using the ROH approach.

An intriguing observation in this finding was the overlap of selective regions in Kazakh Tobet and Kazakh Tazy dogs, both identified by ROH analysis. Specifically, on CFA 18 eROHi in Kazakh Tobets was detected in the range of 3319–4837 kb, while eROHi in Kazakh Tazy spans a similar range of 3319–4393 kb [38]. This region contains genes whose functional roles are not yet fully elucidated. In addition, the *MBP* gene, which showed a selection signal in the ROH analysis on CFA 1 in the Kazakh Tobet, was also identified as a target of selection in several studies involving non-LGD breeds [39, 40]. The central role of *MBP* in the maintenance of myelin [41], together with *CNTNAP2* and *GALR1* — genes involved in neurodevelopment and synaptic plasticity [42–45] that also showed a positive signal in our ROH analysis — supports the hypothesis that cognitive function may have played a significant role in the evolutionary process of dog domestication and the formation of the different breeds [39, 40, 46]. Moreover, these genes may play a role in cross-species domestication processes, as members of the contactin gene family (CNTN) have frequently been identified as selection targets not only in dogs, but also in pigs, sheep, and goats [47, 48].

The two haplotype-based methods further support the significant role of selection on genes related to brain function and behavior in Kazakh Tobet dogs. A significant proportion of these genes were associated with neuronal adhesion, synaptic plasticity, and ion channel function, all of which are important for cognitive flexibility and sensory perception. Among these, the *DCTN1* and *TRIO* genes overlapped with those showing selection signals in European LGD breeds [5]. *DCTN1* is a key component of the dynactin complex and plays an essential role in intracellular transport and motor neuron function [49, 50]. *TRIO*, a Rho-GTPase exchange factor, regulates cell signaling and serves as a central mediator in neuronal development, including processes such as axon guidance and synaptic plasticity [51, 52]. This gene, along with *ITCH*,* NCKAP5*,* PBX3*,* RIMS4*, and *TAF4B*, all of which show selection signals in the Kazakh Tobet, was also reported under selection in a genomic study of 15 indigenous Chinese dog breeds [53]. The *NCKAP5* gene, which is thought to be involved in microtubule bundle formation and microtubule depolymerization in humans, was also under selection pressure in modern British and Swedish dog breeds [46]. In addition, this gene was identified as a potential candidate for temperament traits in cattle [54]. *PBX3*, a transcription factor, is thought to be involved in several processes, including embryonic organ development, eye development, and nervous system development, while *RIMS4* regulates synaptic vesicle recycling, neurotransmitter release, and synaptic plasticity [55, 56]. The two remaining genes, *ITCH* and *TAF4*, are crucial for immune regulation. *ITCH* was found, like *NCKAP5*, to be under selection in modern British and Swedish dog breeds [46].

Similarly, in our analysis of candidate genes, we identified the glutamate receptor genes *GRIK2* and *GRIA1*, the latter of which was also observed in a selection scan in cats [57]. Selective pressure on glutamate receptor genes has been demonstrated in other studies in dogs (*GRIK3* gene) [40] and in cattle [58]. It has been suggested that these genes may be associated with a general trend toward reduced fear in domesticated species [40, 59]. This hypothesis is indirectly supported by the fact that the major candidate genes for neurological behavior in rats do not overlap with these genes [60]. These comparisons also suggest that while positive selection during domestication targets specific pathways, such as the glutamate receptors, it does not necessarily act on the same genes within these pathways in different species [40]. Interestingly, *GRIA1*, along with another of our candidate genes, *EFEMP1*, was also associated with body size and the length-to-height ratio in a previous GWAS analysis conducted in a non-LGD dog breed [61]. Finally, another interesting group of candidate genes in our analysis includes four genes from the solute carrier (SLC) family: *SLC25A26*,* SLC25A4*,* SLC44A3*, and *SLC4A4.* Studies have shown that genes within this family are subject to evolutionary selection in dogs [62] and correlate with aggressive behavior [63–66]. Historically, Kazakh Tobets were subject to strict selection against aggressive behavior toward their owners and community members. This strict selection probably explains why selection pressure has acted on four genes from this family in the breed.

GO and KEGG analysis further confirmed the importance of neuronal and synaptic processes in the evolutionary history of Kazakh Tobet dogs. In addition, several metabolic pathways were enriched, including the regulation of lipolysis in adipocytes. Lipolysis — the breakdown of stored fats into free fatty acids for energy production — is particularly relevant during sustained physical activity and periods of high energy demand [67, 68]. It is plausible that metabolic efficiency and endurance were among the traits selected for in the Kazakh Tobet to support their performance as LGDs in harsh and resource-limited conditions. In addition, the enrichment of signalling pathways involved in circadian control indicates the involvement of biological rhythms in the evolution of the breed. For Kazakh Tobet dogs, maintaining a synchronized internal clock may be critical to their ability to remain alert during certain times of the day or night, optimizing their efficiency in challenging environments. Pathways related to pyrimidine metabolism and neutrophil extracellular trap formation suggest that genetic adaptation in these dogs may have been influenced by immune system functions. Overall, these results suggest that neuronal, metabolic, and immunological functions were important selection factors in the evolution of Kazakh Tobet dogs.

### Limitations

Despite our efforts to create representative samples, the dataset shows an uneven distribution of samples among the different breeds, which may influence the observed genetic patterns. In addition, the samples of Kazakh Tobet dogs were not evenly distributed across regions. The historical prevalence of nomadic pastoralism and breeding of Kazakh Tobets in South and North Kazakhstan explains the higher representation of these regions in our study. Nevertheless, future efforts should focus on achieving a more balanced representation from other regions to more accurately capture the overall genetic diversity of the breed. In addition, merging multiple datasets from different sources resulted in common datasets with a lower number of variants, which may have affected the overall resolution of the analyses. The relationships we observed between breeds are consistent with previously published findings for LGD and non-LGD breeds [1, 69], which is indirect evidence of the reliability of our analyses. Nevertheless, future studies on the genetic relatedness of the Kazakh Tobet with other breeds and populations could benefit from further improvements in data integration and the formation of more balanced datasets. Furthermore, while SNP arrays provide valuable insights into genetic variation, they cannot detect structural variation that may contribute to the observed genetic patterns. Genetic data are used in this study to make inferences about historical gene flow and admixture; however, detailed historical records are needed to support these findings with direct evidence. Finally, the analysis of selection signals focuses on genome-wide SNP variation but does not incorporate functional genomic approaches, such as gene expression studies, that could provide deeper insights into the adaptive significance of genetic variants in Kazakh Tobet dogs.

## Conclusions

Overall, our study suggests that Kazakh Tobet dogs, like other LGD breeds, have a complex genetic heritage characterized by ancient and recent interactions with other breeds and populations. Kazakh Tobet dogs exhibit high genetic diversity and show low differentiation from other LGD breeds. Their genetic profile reveals not only deep connections with Central and West Asian LGDs, but also evidence of introgression with European LGDs, including breeds from the geographically distant Iberian Peninsula. This finding is consistent with historical evidence suggesting that Steppe dogs contributed to the development of Western European breeds. Taken together, these results confirm the central role of human migration and livestock management practices in shaping the genetic diversity of LGDs but may also reflect more recent cases of deliberate crossbreeding. In addition, our results suggest the intriguing possibility that Kazakh Tobets may have evolved within the Southeast Asian lineage, with substantial gene flow from Arctic dog breeds. The genetic clustering of Kazakh Tobets with free-ranging dogs reinforces the idea that evolutionary pressure in open breeding systems, such as those used for LGDs, may not compromise essential functional traits. At the same time, detectable gene flow from European molossoid breeds exposes vulnerabilities that could threaten the integrity of the breed. The genetically isolated group from North Kazakhstan, characterized by high inbreeding, the absence of European molossoid admixture, and clear differentiation from other Kazakh Tobet dogs, should be further studied to better understand its origins, genetic health, and potential role in the preservation of the breed. Selection signatures in genes associated with cognition, behavioral regulation, and physiological adaptation suggest that the breed was shaped by pressures favoring intelligence, balanced temperament, endurance, and immune resistance in the harsh Steppe environment. Taken together, these results provide insights into the evolutionary history, functional adaptation, and current challenges facing the preservation of this unique LGD breed.

## Methods

### Sample collection and DNA extraction

For this study, the genetic material of 75 Kazakh Tobet dogs was collected by non-invasive cheek swabs and/or blood samples. Since there is currently no official, centralized registry for Kazakh Tobet dogs, unregistered dogs were included in the study. The dogs were selected based on an expert evaluation by dog specialists from “Kansonar,” an organization associated with the management of Kazakh national dog breeds. The experts assessed the dogs’ conformity with the official breed standard, which was approved by the Ministry of Ecology and Natural Resources of the Republic of Kazakhstan on March 30, 2023 (https://adilet.zan.kz/rus/docs/V2300032180#z15). The evaluation criteria were based on a comprehensive personal assessment conducted by the experts at various dog shows, special events, and expeditions. The evaluation focused on morphometric measurements, body proportions, temperament, and other parameters defined in the breed standard. In addition, attention was paid to some morphological differences due to the different regions and climatic conditions in which the dogs were bred. Due to the endangered status of the Kazakh Tobet breed, the lack of structured breeding programs, and the decline of herding, there were few working dogs and dogs with high scores. Therefore, dogs with lower scores were also included to represent the genetic diversity of the breed.

The collected blood and buccal epithelial samples were transported in a portable cool box and stored at − 20 °C prior to DNA extraction. Genomic DNA was extracted using the QIAamp DNA kit (Qiagen, MD, USA) according to the manufacturer’s protocol. In addition, 107 samples from a previous study were included [10]. Together with the newly collected samples, a total of 182 samples of Kazakh Tobet dogs (87 females and 95 males) from five regions were used for this study: 106 samples from South Kazakhstan, 52 samples from North Kazakhstan, 16 samples from East Kazakhstan, 5 samples from West Kazakhstan, and 3 samples from Bayan-Ulgii in Mongolia, a district inhabited by ethnic Kazakhs. Genetic material was also collected in this study from one Bankhar, also known as Mongolian Shepherd dog, an LGD breed from Mongolia, and eight Taigan dogs, a sighthound breed from Kyrgyzstan. Images and detailed information on the samples, including the dog code, sex, regions of origin, and cynological assessment values, can be found in Additional file 3: Fig. S1 and Additional file 1: Table S7, respectively.

### SNP genotyping and data preparation

A total of 182 Kazakh Tobet dogs, eight Taigan dogs, and one Bankhar dog were genotyped using a Canine HTS and HD Genotyping BeadChip (Illumina). Genotyping was performed on the iScan system (Illumina) using GenomeStudio software (Illumina), and the data were exported in PLINK v1.9 format (www.cog-genomics.org/plink/1.9/) [70].

In addition to the newly genotyped data, previously published SNP array data from gray wolves, Kazakhstan Shepherd dogs (Kazakh Tobet dogs from Xinjiang) [53] and other LGD and non-LGD breeds, as well as free-ranging (village) dogs were included in this study (Table 4).

**Table 4:** The sources of publicly available SNP data

Source	Identifier	Chip
This study	10.17605/OSF.IO/CSJDP	Canine HTS Genotyping BeadChip (1–zapusk, 2–zapusk, 3–zapusk); CanineHD Genotyping BeadChip (4-zapusk)
Coutinho-Lima et al. 2024 [1]	https://osf.io/qp9eg/?view_only=8638cd71a4024e0aba66c40521661e7f [71]	CanineHD Genotyping BeadChip
Pilot et al. 2015 [72]	https://datadryad.org/stash/dataset/10.5061/dryad.078nc [73]	CanineHD Genotyping BeadChip
Shannon et al. 2015 [8]	https://datadryad.org/stash/dataset/10.5061/dryad.v9t5h [74]	CanineHD Genotyping BeadChip
Yang et al. 2019 [53]	https://datadryad.org/stash/share/u4FKRNZ4wueHyQEnYTeZ59XAWvuVg-aFMhTwqr1gfB4 [75]	CanineHD Genotyping BeadChip
Perfilyeva et al. 2023 [76]	https://osf.io/5shwu/ [77]	CanineHD Genotyping BeadChip

### SNP data processing and quality control

The SNP data were analyzed with PLINK v1.9 [70] in a multi-step process to ensure high data quality for the subsequent genetic analyses. The datasets listed in Table 4 were used to create three datasets: (i) Kazakh Tobet, (ii) LGD, and (iii) LGD, non-LGD, and free-ranging dogs combined. The average call rate in the Kazakh Tobet dataset was 0.976. The number of input and output SNPs and samples, and the filters applied to each dataset are summarized in Table 5.

**Table 5:** Dataset filtering summary and sample information

Dataset	Input SNP after merge	Input sample	Applied filters	Output SNP	Output sample
Kazakh Tobet	173,438	182	–geno 0.01 –hwe 1e-6 –maf 0.05 –not-chr X,Y, MT –indep-pairwise 50 5 0.2 –mind 0.1 Pi_HUT > 0.25	59,237	90
LGD	176,342	180	–geno 0.01 –maf 0.05 –not-chr X,Y,MT –indep-pairwise 50 5 0.2 –mind 0.1 Pi_HUT > 0.5 dataset balance	48,082	148
LGD, non-LGD and free-ranging dogs	176,342	6384	–geno 0.01 –maf 0.05 –not-chr X,Y,MT –indep-pairwise 50 5 0.2 –mind 0.1 Pi_HUT > 0.5 dataset balance	44,305	2456

The following filters were applied to all datasets: missing genotype call rates (–geno), minor allele frequency (–maf), linkage disequilibrium pruning (–indep-pairwise), exclusion of sex chromosomes (–not-chr X,Y,MT), individual-level missing call rates (–mind), and removal of related individuals based on pairwise identity-by-descent estimates (–genome, Pi_HAT). The HWE filter was excluded for the LGD and LGD, non-LGD and free-ranging dog datasets, as the genetic structure of dogs from heterogeneous populations may not meet the assumptions of Hardy–Weinberg equilibrium. In addition, a more permissive Pi_HAT threshold (0.5) was applied in the combined datasets to balance data quality with breed diversity, recognizing that elevated relatedness may reflect shared ancestry within breeds rather than recent familial relationships.

In order to enhance the genetic representativeness of the LGD and LGD, non-LGD and free-ranging dog datasets, a balanced sampling was ensured through the implementation of additional measures. In particular, within the LGD dataset, efforts were focused on reducing the over-representation of Kazakh Tobet dogs, which made up most of the dataset. A maximum of four Kazakh Tobet dogs were selected from each region, with preference given to dogs with the highest expert ratings (Additional file 1: Table S7). Breeds with 20 or fewer individuals were fully represented in the datasets for LGD, non-LGD, and free-ranging dogs. For breeds and free-ranging dogs representing more than 20 individuals, clustering was performed in PCA using the first three principal components with k-means clustering (*k* = 20). One representative individual from each cluster closest to the cluster centroid was retained. This ensured that each breed and free-ranging dog population was represented in the final dataset with a minimum of 3 and a maximum of 20 individuals per breed or population.

The breakdown of samples by dataset can be found in Additional file 1: Table S8. The geographical origin of the LGD and non-LGD breeds is detailed in Additional file 1: Table S9. The family identifier (FID) and individual identifier (IID) for all samples used in this study are provided in Additional file 1: Table S10.

### Genetic diversity analysis

Datasets of Kazakh Tobet and LGD were analyzed to evaluate the genetic diversity and polymorphism within the LGD breeds. The number of genotypes and Hardy–Weinberg equilibrium statistics were generated using the PLINK v1.9 –hardy command, while allele frequencies were calculated using the –freq command. This data was then used to calculate Ho, He, PIC, and Fis. He was calculated based on the allele frequencies in Hardy‒Weinberg equilibrium. The PIC was calculated as the difference between the total genetic variability of a locus and the probability of random allele matches. Fis was calculated as the difference between He and Ho, divided by He. Positive values indicated inbreeding; negative values indicated excessive heterozygosity.

### Principal component analysis

PCA of all three datasets was performed using PLINK v1.9 with the –pca command. The resulting data were visualized in R using the package ggplot2 [78].

### Admixture analysis

Admixture analysis was performed for all three datasets using the ADMIXTURE software [79]. A cross-validation procedure was used to determine the optimal number of population clusters (*K*), where the optimal K was identified as the one that gave the lowest CV error. For the Kazakh Tobet dataset, a range of K values from 2 to 10 was tested, with 10 cross-validations performed for each K. For the LGD dataset and the combined dataset of LGD, non-LGD and free-ranging dogs, the range of K values tested corresponded to the number of breeds in each dataset, with 10 cross-validations performed for each K. The admixture plots were visually inspected to identify clusters and assess population structure. The resulting data were visualized in R using the package ggplot2 [78].

### Phylogenetic analyses and gene flow

D-statistics for the LGD dataset, as well as for the combined dataset of LGD, non-LGD and free-ranging dogs were calculated using Dsuite v0.5r58, with the gray wolf population serving as the outgroup [80]. A filtering threshold based on the *p*-value with Bonferroni correction and a *Z*-score > 5 was applied to identify statistically significant signals of gene flow. The resulting data were visualized in R using the packages ggplot2 [78], dplyr [81], and ggrepel [82]. The patterns of divergence and gene flow for the LGD dataset were also inferred using the maximum likelihood algorithm in TreeMix v1.13 [83]. A phylogenetic network was created by sequentially including up to 20 migration events and the population of gray wolves, which served as the root group. The optimal number of migration edges was determined using the elbow method by evaluating the changes in log-likelihood values across migration events. The results were visualized using the R script integrated into the software.

An NJ tree for the combined dataset if LGD, non-LGD, and free-ranging dogs was created by calculating genomic distances using PLINK v1.9 with the –distance command applied to the filtered dataset. The resulting distance matrix was processed in R to create an NJ-tree with the function nj from the package ape [84]. The gray wolf sample was selected as the outgroup for rooting the tree. The rooted tree was visualized using iTOL (Interactive Tree of Life) v6 [85].

### Identification of signatures of selection

ROH, iHS and nSL were used to identify selection signals in the Kazakh Tobet dataset. The original unfiltered dataset, which included 182 dogs and 173,438 SNPs, was filtered as described in Table 5. However, no filtering based on minor allele frequency (–maf), Hardy–Weinberg equilibrium (–hwe), or linkage disequilibrium pruning (–indep-pairwise) was performed to avoid the potential exclusion of weaker selection signals [86]. As a result, 90 dogs and 157,248 variants passed the filter criteria and were used as input for the analysis of the selection signals.

The ROH analysis was performed using a sliding window approach implemented in PLINK v1.9 with the –homozyg command. The values of the parameters were selected based on standard values that are commonly used for this analysis [87]. A sliding window of 50 SNPs (–homozyg-window-snp 50) was used to identify continuous homozygous regions. The following criteria were used: a minimum of 50 SNPs per segment (–homozyg-snp 50), a minimum length of 1 Mb (–homozyg-kb 100) and a maximum allowed distance of 1 Mb between consecutive SNPs (–homozyg-gap 1000). The analysis allowed up to one heterozygous call (–homozyg-window-het 1) and up to two missing genotypes (–homozyg-window-missing 2). Graphical representations of ROH were generated using the R package detectRUNs [88]. A threshold of 50% was used to identify the eROHi across the population. The inbreeding coefficient FROH was determined by calculating the proportion of the genome covered by ROH.

The nSL and iHS methods were applied to phased haplotypes. Haplotypes were phased using Beagle v5.5 [89]. The nSL and iHS scores were implemented in Selscan [90] and converted to a two-sided *p*-value: *p* = − log10[1–2|Φ(iHS or nSL) − 0.5|], where Φ(iHS or nSL) is the cumulative Gaussian distribution function of iHS or nSL. A SNP was considered to be under selection if it exceeded the significance threshold of − log10(*p*-value) = 4.

SNPeff [91] was used to obtain the annotations of SNPs and genes in eROHi based on the CanFam3.1 (GCF_000002285.5) genome assembly. GO analysis and KEGG pathway analysis were performed for the genes of eROHi using the R package clusterProfiler and the org.Cf.eg.db database [92], with filtering based on a *q*-value ≤ 0.05 for GO analysis and a *p*-value ≤ 0.05 for KEGG analysis.

## Supplementary Information

Additional file 1: Table S1 SNP-based genetic variability in LGD breeds. Table S2 Introgression events among LGD breeds based on D-statistics. Table S3 FROH of Kazakh Tobet dogs. Table S4 Annotation of SNP identified in ROH, iHS and nSL tests. Table S5 Characterization of genes with known functional significance identified in ROH, iHS and nSL tests. Table S6 GO and KEGG annotation of genes identified in ROH, iHS and nSL tests. Table S7 Kazakh Tobet sample data. Table S8 Sample distribution by dataset. Table S9 Geographic origin of LGDand non-LGD breeds. Table S10 Sample Information: FID, IID and source

Additional file 2: Fig. S1 Genetic structure of Kazakh Tobet dogs based on a reduced dataset (maximum 5 dogs per region). a PCA plot. b Cross-validation (CV) errors for different K values. c Admixture plot for the best K = 2. Fig. S2 A detailed PCA plot of the LGD and non-LGD breeds and free-ranging dogs. Fig. S3 Comparative genetic structure of Kazakh Tobet dogs and non-LGD breeds. a, c, e Cross-validation (CV) errors across different K values. b Admixture plot showing genetic structure of Kazakh Tobet dogs in comparison with Central Asian and Arctic non-LGD breeds at the optimal K values. d Admixture plot of Kazakh Tobet dogs, African and East Asian non-LGD breeds at the optimal K values. f Admixture plot of Kazakh Tobet dogs and molossoid non-LGD breeds, including individuals from North Kazakhstan (TB198–TB208), which showed a distinct genetic profile in both PCA and admixture analyses

Additional file 3: Fig. S1 Pictures of the examined Kazakh Tobet dogs

## Data Availability

The genotype data generated in this study have been deposited in an Open Science Framework repository and are available at: 10.17605/OSF.IO/CSJDP. All publicly available datasets used in this article and supporting the conclusions of this study are fully referenced in the manuscript and listed in the reference list, along with their respective DOIs. An overview table summarising all datasets, including chip type, source, and identifier, is provided in Table 4 of this manuscript.

## Abbreviations

LGD: Livestock guarding dog
SNP: Single nucleotide polymorphism
WES: Whole exome sequencing
Ho: Observed heterozygosity
He: Expected heterozygosity
PIC: Polymorphism information content
Fis: Inbreeding coefficient
PCA: Principal component analysis
CV error: Cross-validation error
SD: Standard deviations
NJ tree: Neighbor-joining tree
ROH: Runs of homozygosity
iHS: Integrated haplotype score
nSL: Number of segregating sites by length
IBD: Identity by descent
CFA: *Canis familiaris* autosome
FROH: ROH-based inbreeding coefficients
eROHi: Extreme runs of homozygosity islands
GO: Gene Ontology
KEGG: Kyoto Encyclopedia of Genes and Genomes
CNTN: Contactin gene family
FID: Family identifier
IID: Individual identifier
