# Supplementary material for: Kazakh Tobet dogs in the genomic landscape: refining the history of livestock guardian breeds
Source: BMC Biol. 2025 Aug 5;23:240. doi: 10.1186/s12915-025-02344-2 (PMC12326758; doi:10.1186/s12915-025-02344-2)

Additional file 2: Fig. S1 Genetic structure of Kazakh Tobet dogs based on a reduced dataset (maximum 5 dogs per region). **a** PCA plot. **b** Cross-validation (CV) errors for different K values. **c** Admixture plot for the best K = 2

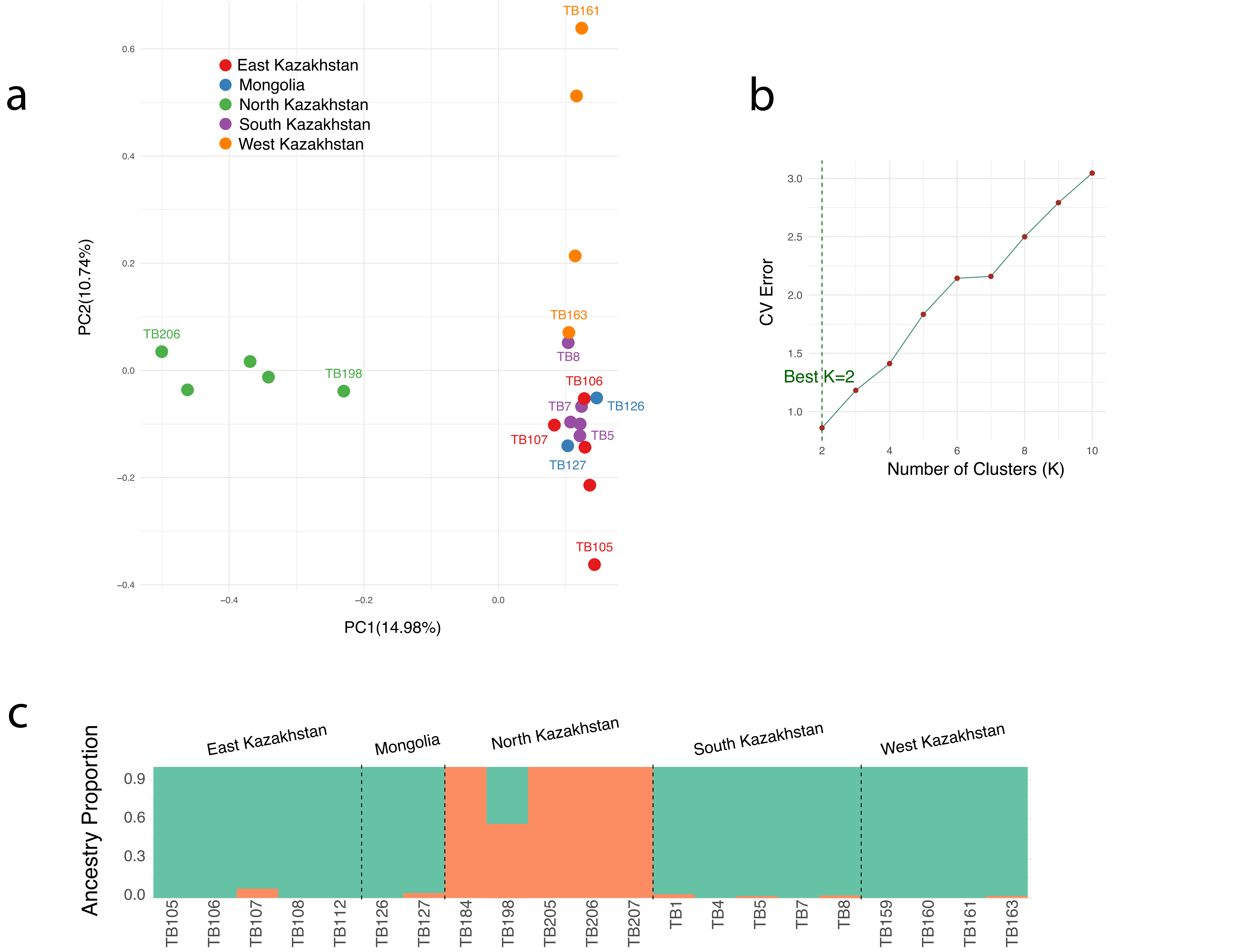

Additional file 2: Fig. S2. A detailed PCA plot of the LGD and non-LGD breeds and free-ranging dogs

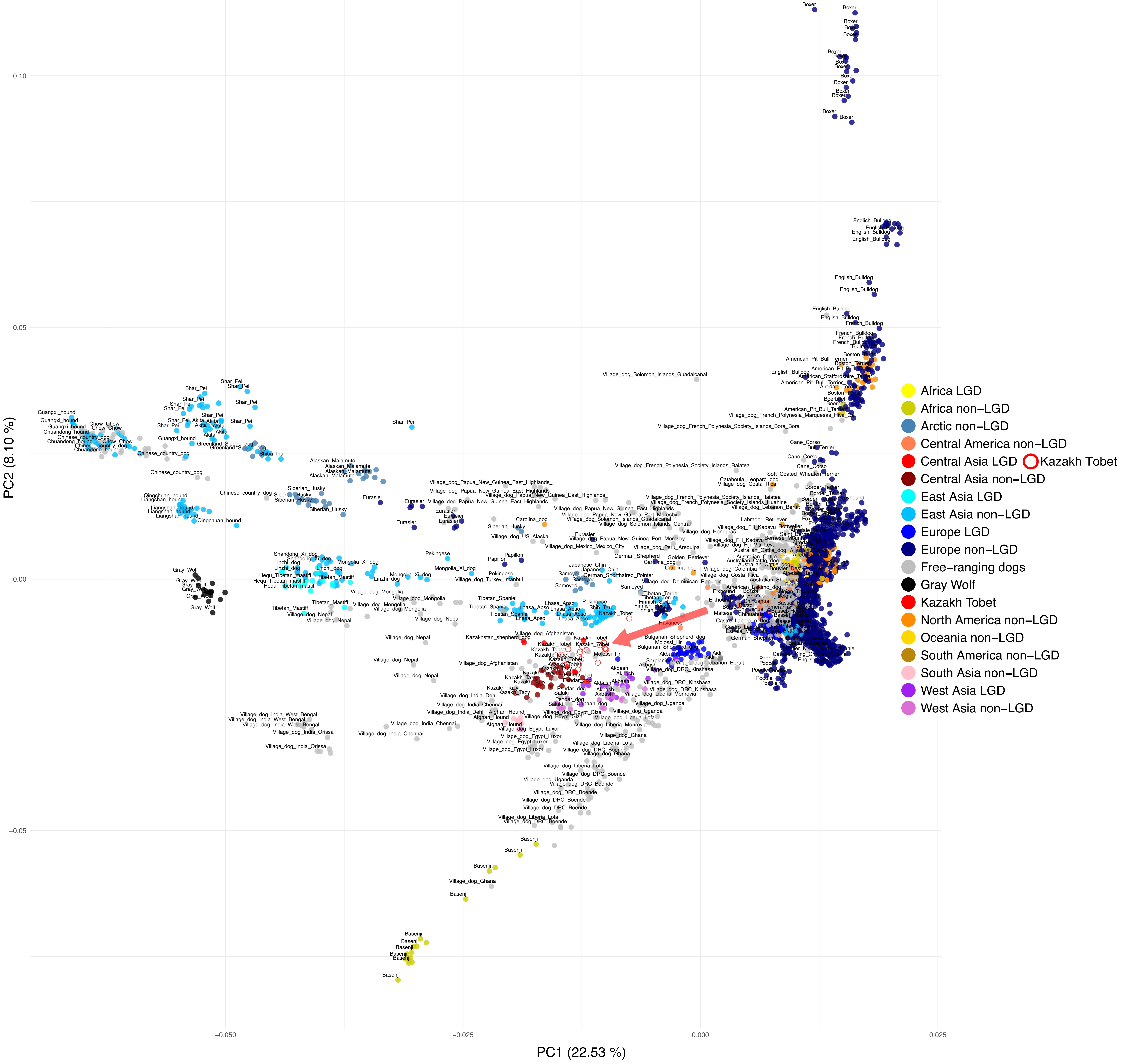

Additional file 2. Fig. S3. Comparative genetic structure of Kazakh Tobet dogs and non-LGD breeds. **a, c, e** Cross-validation (CV) errors across different K values. **b** Admixture plot showing genetic structure of Kazakh Tobet dogs in comparison with Central Asian and Arctic non-LGD breeds at the optimal K values. **d** Admixture plot of Kazakh Tobet dogs, African and East Asian non-LGD breeds at the optimal K values. **f** Admixture plot of Kazakh Tobet dogs and molossoid non-LGD breeds, including individuals from North Kazakhstan (TB198–TB208), which showed a distinct genetic profile in both PCA and admixture analyses

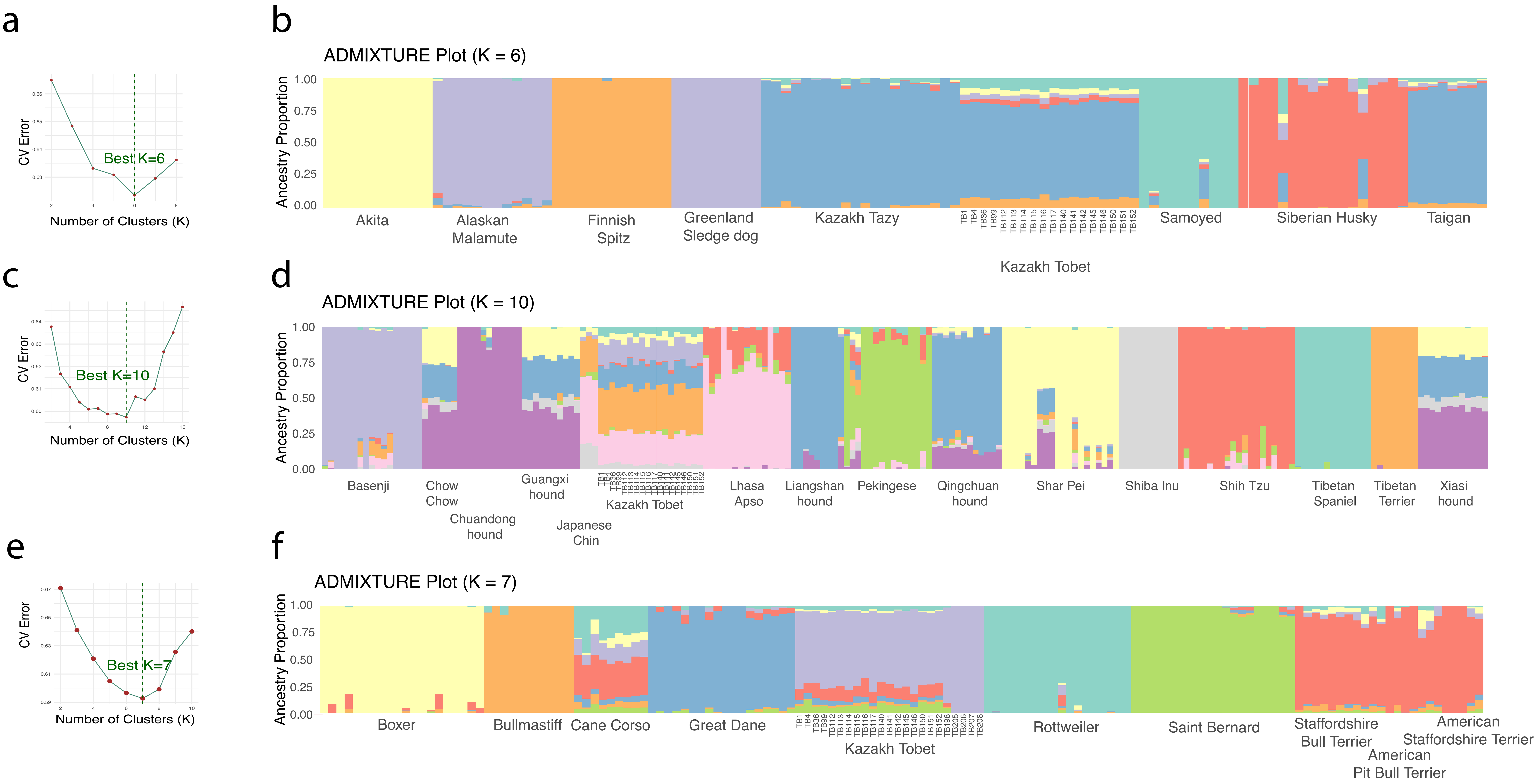

Supplement: Supplementary file 2 — Additional file 2: Fig. S1 Genetic structure of Kazakh Tobet dogs based on a reduced dataset (maximum 5 dogs per region). a PCA plot. b Cross-validation (CV) errors for different K values. c Admixture plot for the best K = 2. Fig. S2 A detailed PCA plot of the LGD and non-LGD breeds and free-ranging dogs. Fig. S3 Comparative genetic structure of Kazakh Tobet dogs and non-LGD breeds. a, c, e Cross-validation (CV) errors across different K values. b Admixture plot showing genetic structure of Kazakh Tobet dogs in comparison with Central Asian and Arctic non-LGD breeds at the optimal K values. d Admixture plot of Kazakh Tobet dogs, African and East Asian non-LGD breeds at the optimal K values. f Admixture plot of Kazakh Tobet dogs and molossoid non-LGD breeds, including individuals from North Kazakhstan (TB198–TB208), which showed a distinct genetic profile in both PCA and admixture analyses [file 12915_2025_2344_MOESM2_ESM.pdf]
