# Supplementary material for: Kazakh Tobet dogs in the genomic landscape: refining the history of livestock guardian breeds
Source: BMC Biol. 2025 Aug 5;23:240. doi: 10.1186/s12915-025-02344-2 (PMC12326758; doi:10.1186/s12915-025-02344-2)

Additional file 3: Fig. S1 Pictures of the examined Kazakh Tobet dogs.

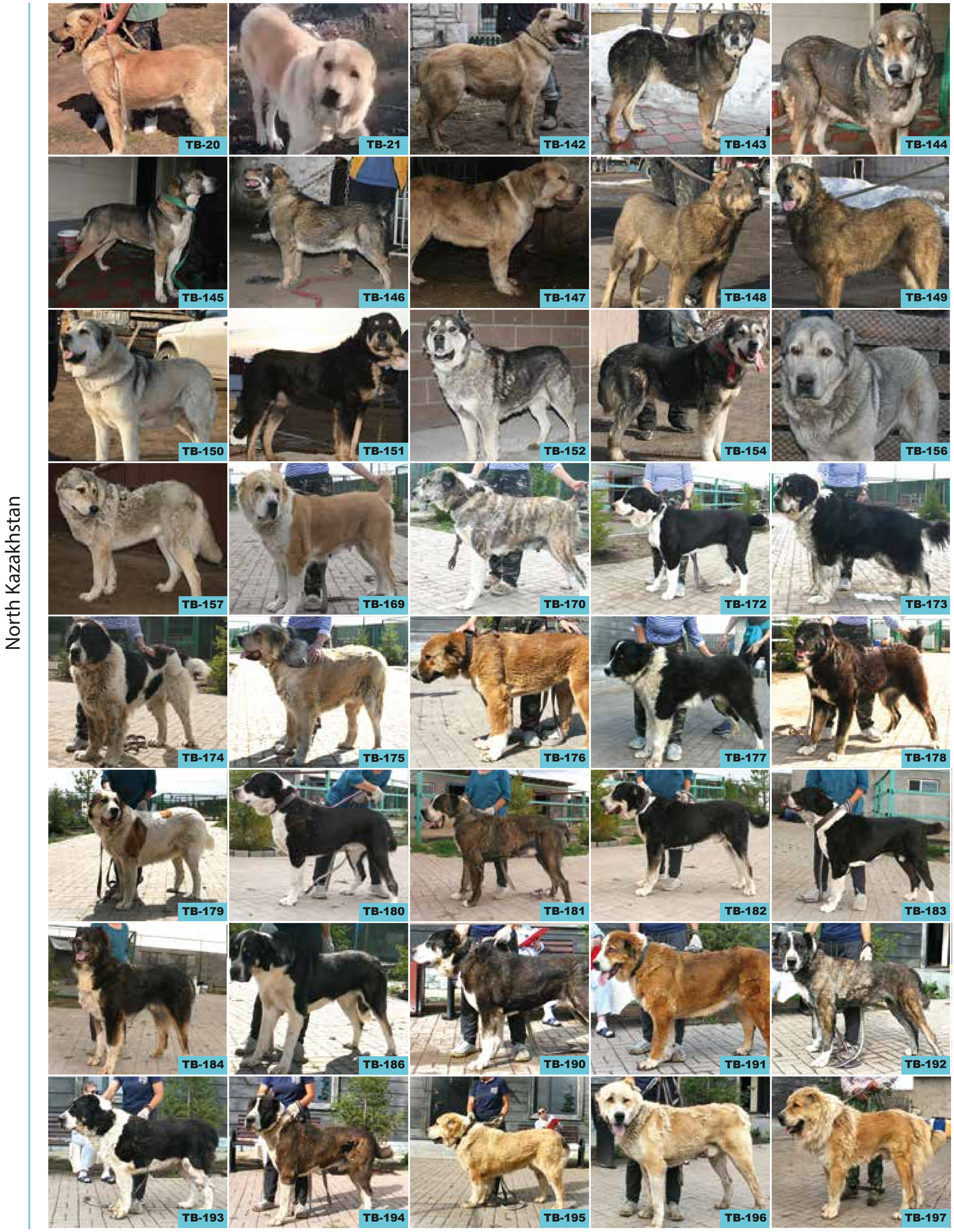

North Kazakhstan

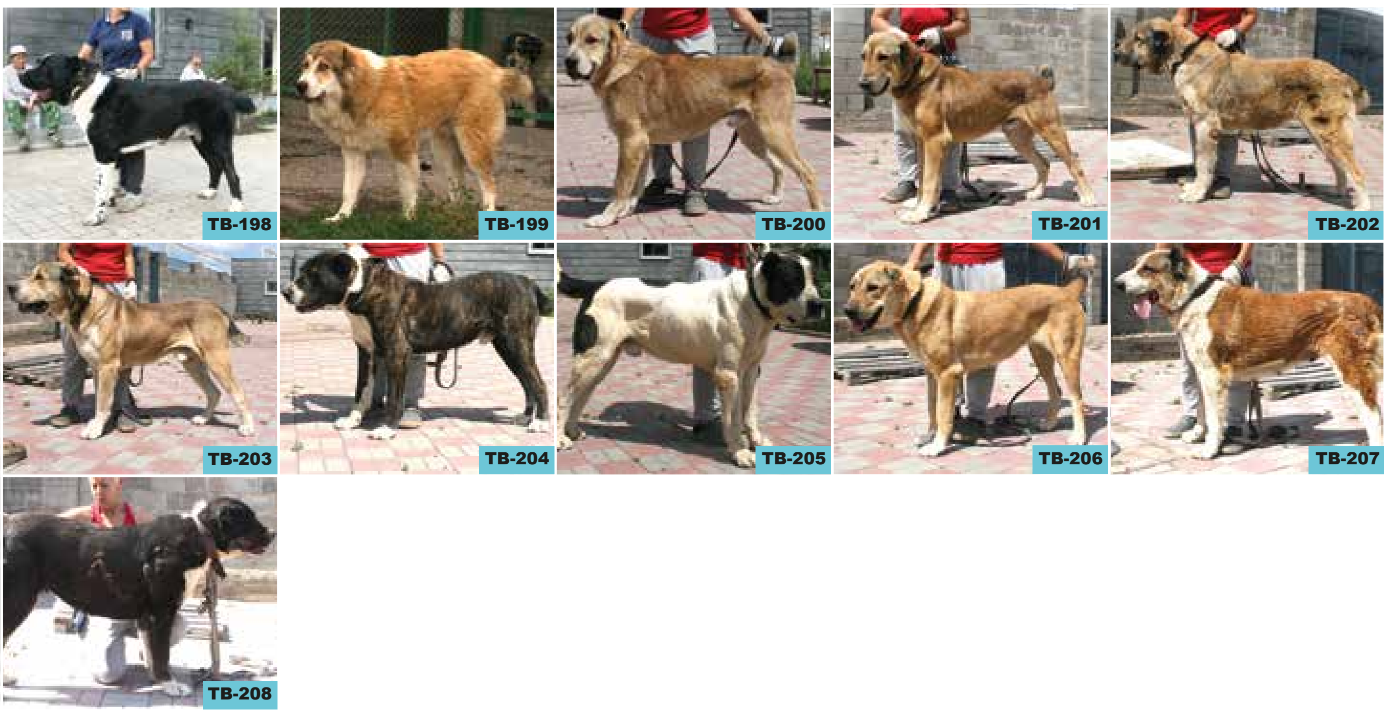

South Kazakhstan

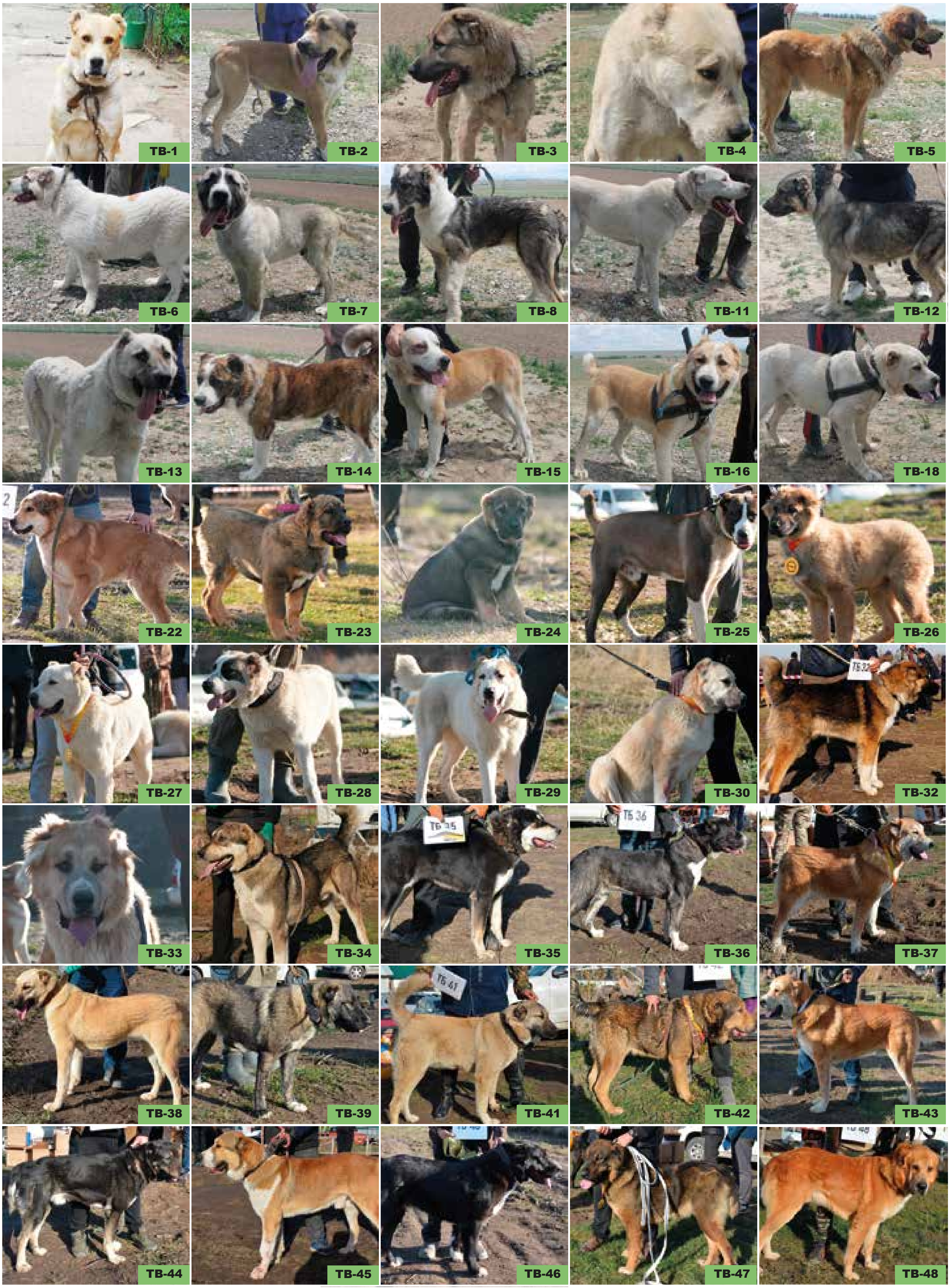

South Kazakhstan

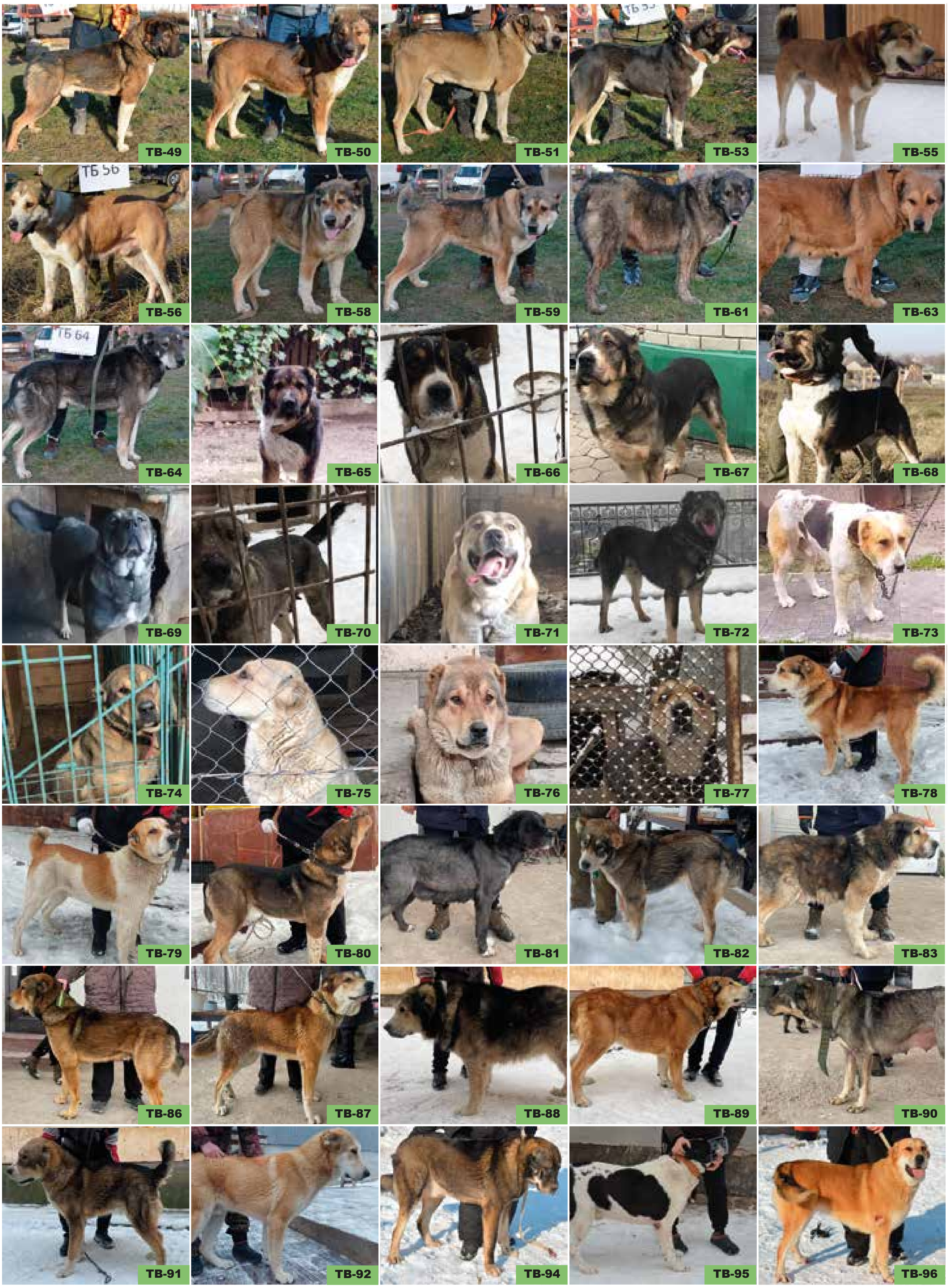

South Kazakhstan

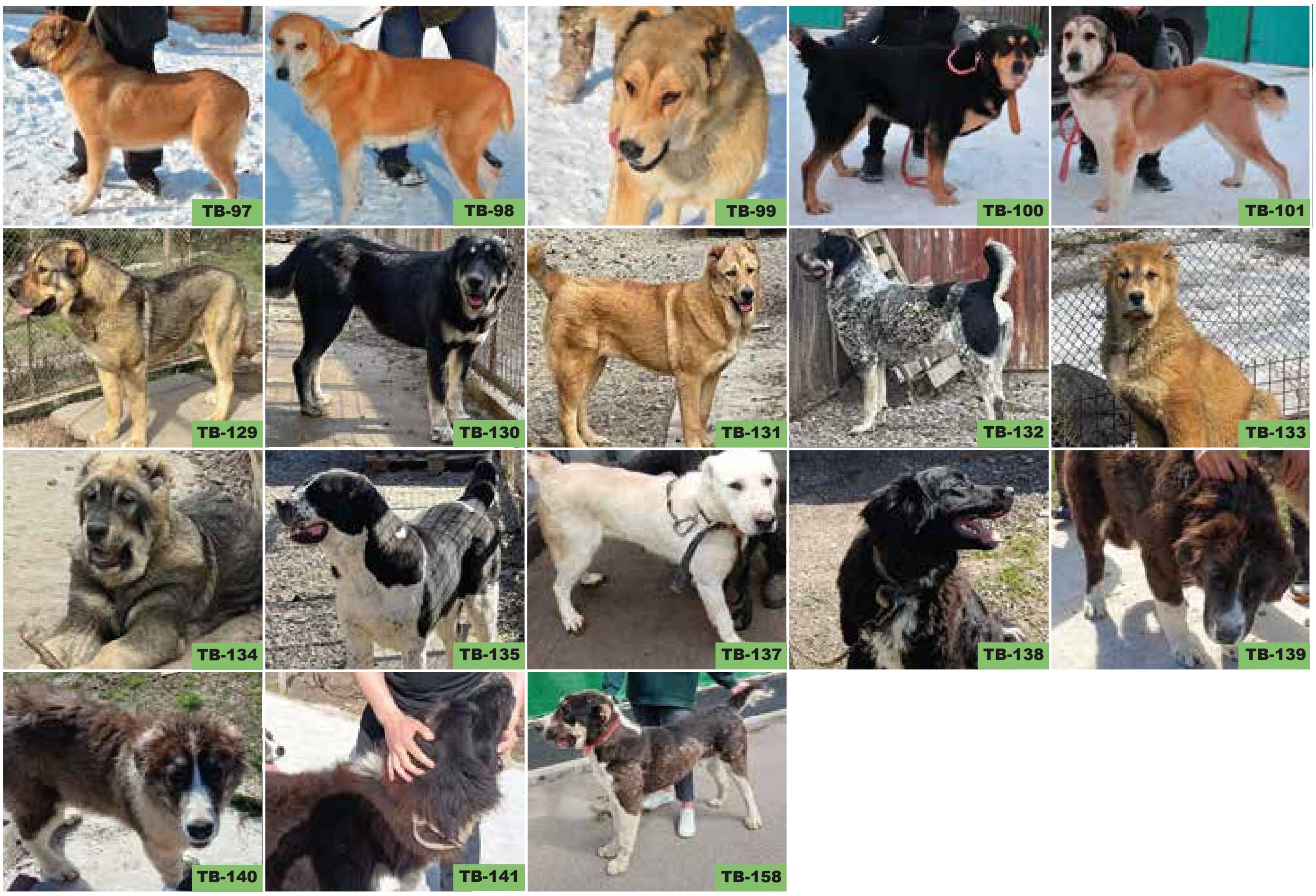

East Kazakhstan

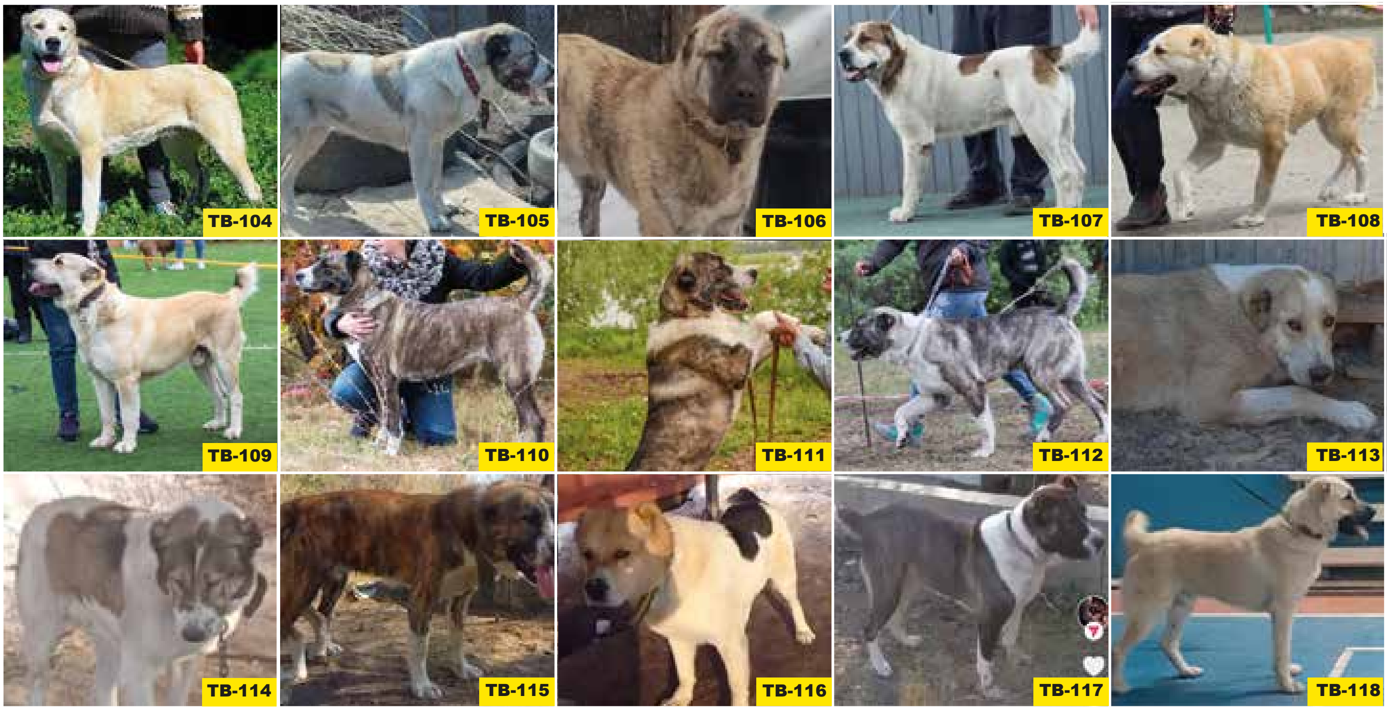

Mongolia

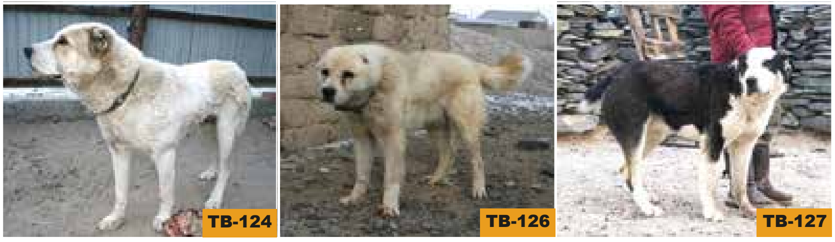

West Kazakhstan

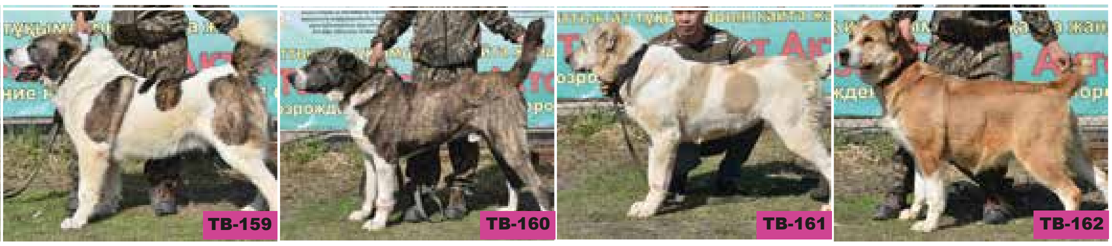

Supplement: Supplementary file 3 — Additional file 3: Fig. S1 Pictures of the examined Kazakh Tobet dogs [file 12915_2025_2344_MOESM3_ESM.pdf]
